# [μ-Bis(diphenyl­arsanyl)methane-1:2κ^2^ *As*:*As*′]nona­carbonyl-1κ^3^C,2κ^3^C,3κ^3^C-(triisoprop­yl phos­phite-3κ*P*)-*triangulo*-triruthenium(0)

**DOI:** 10.1107/S1600536811012050

**Published:** 2011-04-07

**Authors:** Omar bin Shawkataly, Mohd. Gulfam Alam, Chin Sing Yeap, Hoong-Kun Fun

**Affiliations:** a Chemical Sciences Programme, School of Distance Education, Universiti Sains Malaysia, 11800 USM, Penang, Malaysia; b X-ray Crystallography Unit, School of Physics, Universiti Sains Malaysia, 11800 USM, Penang, Malaysia

## Abstract

The asymmetric unit of the title *triangulo*-triruthenium compound, Ru_3_(CO)_9_(μ-Ph_2_AsCH_2_AsPh_2_)(P[OCH(CH_3_)_2_]_3_) or [Ru_3_(C_25_H_22_As_2_)(C_9_H_21_O_3_P)(CO)_9_], contains two mol­ecules of the *triangulo*-triruthenium complex. The bis­(diphenyl­arsanyl)methane ligand bridges an Ru—Ru bond and the monodentate phosphite ligand binds to the third Ru atom. Both the arsine and phosphite ligands are equatorial with respect to the Ru_3_ triangle. Additionally, each Ru atom carries one equatorial and two axial terminal carbonyl ligands. The dihedral angles between the pairs of benzene rings bound to individual As atoms are 85.67 (8) and 75.91 (8) in the first independent mol­ecule and 74.64 (8) and 70.76 (9) in the second. In the crystal, mol­ecules are linked into a three-dimensional framework by inter­molecular C—H⋯O hydrogen bonds.

## Related literature

For related structures, see: Bruce *et al.* (1983 ▶, 1988 ▶); Churchill *et al.* (1977 ▶); Shawkataly *et al.* (1998 ▶). For the synthesis, see: Shawkataly *et al.* (2011 ▶); Bruce *et al.* (1987 ▶). For the stability of the temperature controller used in the data collection, see: Cosier & Glazer (1986 ▶). 
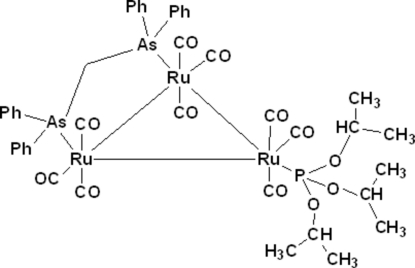


## Experimental

### 

#### Crystal data

[Ru_3_(C_25_H_22_As_2_)(C_9_H_21_O_3_P)(CO)_9_]
*M* *_r_* = 1235.79
Triclinic, 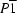

*a* = 12.3481 (3) Å
*b* = 18.1697 (4) Å
*c* = 21.5295 (5) Å
α = 93.494 (1)°
β = 103.230 (1)°
γ = 90.383 (1)°
*V* = 4692.38 (19) Å^3^
*Z* = 4
Mo *K*α radiation
μ = 2.45 mm^−1^
*T* = 100 K
0.35 × 0.23 × 0.19 mm

#### Data collection

Bruker APEXII DUO CCD area-detector diffractometer
Absorption correction: multi-scan (*SADABS*; Bruker, 2009 ▶) *T*_min_ = 0.483, *T*_max_ = 0.659
161249 measured reflections
40931 independent reflections
35153 reflections with *I* > 2σ(*I*)
*R*_int_ = 0.026

#### Refinement

*R*[*F* ^2^ > 2σ(*F* ^2^)] = 0.024
*wR*(*F* ^2^) = 0.055
*S* = 1.01
40931 reflections
1093 parameters
H-atom parameters constrained
Δρ_max_ = 1.57 e Å^−3^
Δρ_min_ = −1.26 e Å^−3^

### 

Data collection: *APEX2* (Bruker, 2009 ▶); cell refinement: *SAINT* (Bruker, 2009 ▶); data reduction: *SAINT*; program(s) used to solve structure: *SHELXTL* (Sheldrick, 2008 ▶); program(s) used to refine structure: *SHELXTL*; molecular graphics: *SHELXTL*; software used to prepare material for publication: *SHELXTL* and *PLATON* (Spek, 2009 ▶).

## Supplementary Material

Crystal structure: contains datablocks global, I. DOI: 10.1107/S1600536811012050/sj5121sup1.cif

Structure factors: contains datablocks I. DOI: 10.1107/S1600536811012050/sj5121Isup2.hkl

Additional supplementary materials:  crystallographic information; 3D view; checkCIF report

## Figures and Tables

**Table 1:** Hydrogen-bond geometry (Å, °)

*D*—H⋯*A*	*D*—H	H⋯*A*	*D*⋯*A*	*D*—H⋯*A*
C10*B*—H10*B*⋯O6*A*^i^	0.93	2.59	3.293 (2)	132
C17*B*—H17*B*⋯O2*B*^ii^	0.93	2.57	3.477 (2)	165
C33*A*—H33*B*⋯O2*A*^iii^	0.96	2.56	3.492 (2)	164

Symmetry codes: (i) 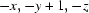; (ii) 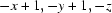; (iii) 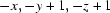.

**Fig. 1.**
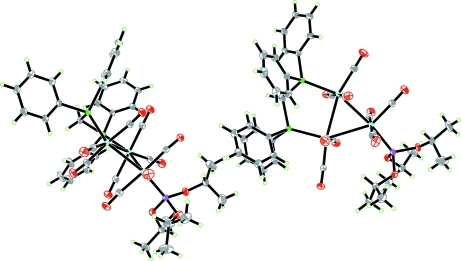
The asymmetric unit, showing 50% probability displacement ellipsoids.

**Fig. 2.**
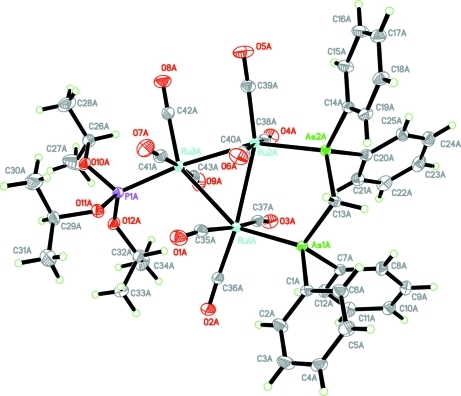
The structure of molecule A, showing 50% probability displacement ellipsoids and the atom-numbering scheme. Atoms in molecule B are similarly labelled.

**Fig. 3.**
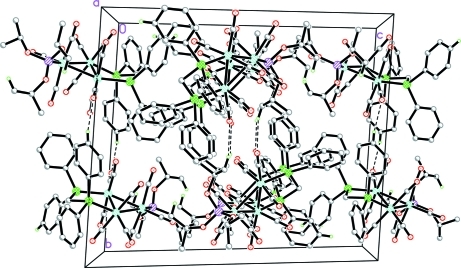
The crystal packing of (I) viewed along the c axis. Dashed lines indicate hydrogen bonds. H atoms not involved in the hydrogen bond interactions have been omitted for clarity.

## supplementary crystallographic information

### Comment

Syntheses and crystal structures of substituted *triangulo*-triruthenium clusters have been of interest to researchers due to structural variations and catalytic activities. A large number of substituted derivatives, Ru_3_(CO)_12-n_L_n_ (L = group 15 ligand) have been reported (Bruce *et al.*, 1988). As part of our ongoing studies (Shawkataly *et al.*, 2011), we report here a mixed-ligand metal-carbonyl cluster complex, Ru_3_(CO)_9_(µ-Ph_2_AsCH_2_AsPh_2_)(P[OCH(CH_3_)_2_]_3_).

The asymmetric unit consists of two crystallographically independent molecules (Fig. 1) of the *triangulo*-triruthenium complex, A and B. Fig. 2 shows the atom numbering scheme for molecule A. Atoms of molecule B were labelled similarly. The bond lengths and angles of the title compound are comparable to those found in its related structure (Shawkataly *et al.*, 1998). The bis(diphenylarsino)methane ligand bridges the Ru1 – Ru2 bond and the monodentate phosphite ligand bonds to the Ru3 atom. Both the phosphite and arsine ligands are equatorial with respect to the Ru_3_ triangle. Additionally, each Ru atom carries one equatorial and two axial terminal carbonyl ligands. The dihedral angles between the two benzene rings (C1-C6/C7-C12 & C14-C19/C20-C25) are 85.67 (8), 75.91 (8) and 74.64 (8), 70.76 (9) for the two diphenylarsanyl groups of molecule A and B, respectively.

In the title compound, one of the Ru – Ru bonds is noticeably longer compared to the other two Ru – Ru bonds. The unevenness in the lengths of Ru – Ru bonds in comparison with those of the Ru_3_(CO)_12_ structure (Churchill *et al.*, 1977), can be attributed to the steric effect induced by the bulky substituent. In the crystal, molecules are linked into a three-dimensional framework by intermolecular C—H···O weak intearctions. (Fig. 3).

### Experimental

All manipulations were performed under a dry, oxygen-free dinitrogen atmosphere using standard Schlenk techniques. All solvents were dried over sodium and distilled from sodium benzophenone ketyl under nitrogen (Bruce *et al.*, 1987). Isopropyl phosphite was used as received and bis(diphenylarsino)methane (Bruce *et al.*, 1983) was prepared by the reported procedure.

Equimolar quantities of Ru_3_(CO)_10_(µ-Ph_2_AsCH_2_AsPh) and isopropyl phosphite were refluxed in THF under nitrogen for 20 minutes. The reaction mixture turned intense red. The solvent was removed under vacuum. The reaction mixture was separated by TLC (dichloromethane:hexane, 1:3). Two bands appeared. The major band (red) R_f _= 0.61 was separated and characterized. The compound was crystallised from CH_2_Cl_2_- CH_3_OH, 1:3 yield = 55.2 %, m.p. 189°C. IR(cyclohexane): v (CO) 2127s, 2059 vs, 2042 s, 2017 s, 1988 s cm^-1^.

### Refinement

All hydrogen atoms were positioned geometrically and refined using a riding model with C—H = 0.93–0.98 Å and U_iso_(H) = 1.2U_eq_(C) and 1.5U_eq_ (C of methyl group).

### Crystal data

**Table:**

[Ru_3_(C_25_H_22_As_2_)(C_9_H_21_O_3_P)(CO)_9_]	*Z* = 4
*M**_r_* = 1235.79	*F*(000) = 2440
Triclinic, *P*1	*D*_x_ = 1.749 Mg m^−^^3^
Hall symbol: -P 1	Mo *K*α radiation, λ = 0.71073 Å
*a* = 12.3481 (3) Å	Cell parameters from 9785 reflections
*b* = 18.1697 (4) Å	θ = 2.8–37.6°
*c* = 21.5295 (5) Å	µ = 2.45 mm^−^^1^
α = 93.494 (1)°	*T* = 100 K
β = 103.230 (1)°	Plate, red
γ = 90.383 (1)°	0.35 × 0.23 × 0.19 mm
*V* = 4692.38 (19) Å^3^	

### Data collection

**Table:**

Bruker APEXII DUO CCD area-detector diffractometer	40931 independent reflections
Radiation source: fine-focus sealed tube	35153 reflections with *I* > 2σ(*I*)
graphite	*R*_int_ = 0.026
φ and ω scans	θ_max_ = 35.0°, θ_min_ = 1.8°
Absorption correction: multi-scan (*SADABS*; Bruker, 2009)	*h* = −19→19
*T*_min_ = 0.483, *T*_max_ = 0.659	*k* = −29→29
161249 measured reflections	*l* = −34→34

### Refinement

**Table:**

Refinement on *F*^2^	Primary atom site location: structure-invariant direct methods
Least-squares matrix: full	Secondary atom site location: difference Fourier map
*R*[*F*^2^ > 2σ(*F*^2^)] = 0.024	Hydrogen site location: inferred from neighbouring sites
*wR*(*F*^2^) = 0.055	H-atom parameters constrained
*S* = 1.01	*w* = 1/[σ^2^(*F*_o_^2^) + (0.0207*P*)^2^ + 3.4447*P*] where *P* = (*F*_o_^2^ + 2*F*_c_^2^)/3
40931 reflections	(Δ/σ)_max_ = 0.005
1093 parameters	Δρ_max_ = 1.57 e Å^−^^3^
0 restraints	Δρ_min_ = −1.26 e Å^−^^3^

### Special details

**Table:**

Experimental. The crystal was placed in the cold stream of an Oxford Cyrosystems Cobra open-flow nitrogen cryostat (Cosier & Glazer, 1986) operating at 100.0 (1) K.
Geometry. All esds (except the esd in the dihedral angle between two l.s. planes) are estimated using the full covariance matrix. The cell esds are taken into account individually in the estimation of esds in distances, angles and torsion angles; correlations between esds in cell parameters are only used when they are defined by crystal symmetry. An approximate (isotropic) treatment of cell esds is used for estimating esds involving l.s. planes.
Refinement. Refinement of F^2^ against ALL reflections. The weighted R-factor wR and goodness of fit S are based on F^2^, conventional R-factors R are based on F, with F set to zero for negative F^2^. The threshold expression of F^2^ > 2sigma(F^2^) is used only for calculating R-factors(gt) etc. and is not relevant to the choice of reflections for refinement. R-factors based on F^2^ are statistically about twice as large as those based on F, and R- factors based on ALL data will be even larger.

### Fractional atomic coordinates and isotropic or equivalent isotropic displacement parameters (Å^2^)

**Table:**

	*x*	*y*	*z*	*U*_iso_*/*U*_eq_	
Ru1A	0.185622 (9)	0.312340 (6)	0.427544 (5)	0.01368 (2)	
Ru2A	0.340063 (9)	0.193747 (6)	0.432267 (5)	0.01354 (2)	
Ru3A	0.197922 (9)	0.197674 (6)	0.517361 (5)	0.01439 (2)	
As1A	0.236634 (11)	0.355824 (7)	0.331871 (6)	0.01348 (2)	
As2A	0.428726 (11)	0.234754 (8)	0.351165 (6)	0.01387 (2)	
P1A	0.05349 (3)	0.20550 (2)	0.567872 (17)	0.01507 (6)	
O1A	−0.03079 (10)	0.22772 (7)	0.36225 (6)	0.0276 (2)	
O2A	0.03402 (11)	0.43647 (7)	0.44928 (6)	0.0281 (3)	
O3A	0.37695 (11)	0.39986 (7)	0.51876 (6)	0.0268 (2)	
O4A	0.51675 (10)	0.26282 (7)	0.54581 (6)	0.0247 (2)	
O5A	0.43156 (12)	0.03960 (7)	0.44851 (7)	0.0338 (3)	
O6A	0.16098 (10)	0.13352 (7)	0.31667 (6)	0.0269 (2)	
O7A	0.04765 (11)	0.08535 (7)	0.42250 (6)	0.0293 (2)	
O8A	0.32119 (11)	0.06903 (7)	0.58178 (6)	0.0302 (3)	
O9A	0.33184 (11)	0.30623 (8)	0.62211 (6)	0.0303 (3)	
O10A	0.04745 (9)	0.14607 (6)	0.61850 (5)	0.01956 (19)	
O11A	−0.06140 (9)	0.18840 (6)	0.51830 (5)	0.01920 (19)	
O12A	0.03848 (9)	0.27785 (6)	0.61109 (5)	0.01966 (19)	
C1A	0.12125 (11)	0.38267 (7)	0.25888 (7)	0.0154 (2)	
C2A	0.02752 (13)	0.41811 (9)	0.27021 (8)	0.0230 (3)	
H2AA	0.0163	0.4234	0.3115	0.028*	
C3A	−0.04975 (14)	0.44579 (10)	0.21959 (9)	0.0270 (3)	
H3AA	−0.1118	0.4701	0.2274	0.032*	
C4A	−0.03447 (13)	0.43732 (9)	0.15792 (8)	0.0229 (3)	
H4AA	−0.0861	0.4560	0.1244	0.027*	
C5A	0.05729 (14)	0.40114 (10)	0.14619 (8)	0.0258 (3)	
H5AA	0.0667	0.3945	0.1046	0.031*	
C6A	0.13607 (13)	0.37452 (9)	0.19675 (7)	0.0234 (3)	
H6AA	0.1988	0.3512	0.1888	0.028*	
C7A	0.32135 (11)	0.44843 (8)	0.34206 (7)	0.0158 (2)	
C8A	0.38946 (14)	0.46559 (9)	0.30141 (8)	0.0221 (3)	
H8AA	0.4005	0.4307	0.2701	0.027*	
C9A	0.44112 (14)	0.53480 (9)	0.30737 (8)	0.0239 (3)	
H9AA	0.4866	0.5458	0.2801	0.029*	
C10A	0.42536 (13)	0.58731 (9)	0.35355 (8)	0.0222 (3)	
H10A	0.4602	0.6334	0.3574	0.027*	
C11A	0.35700 (14)	0.57061 (9)	0.39414 (8)	0.0231 (3)	
H11A	0.3459	0.6058	0.4252	0.028*	
C12A	0.30522 (13)	0.50151 (8)	0.38852 (7)	0.0195 (2)	
H12A	0.2597	0.4907	0.4158	0.023*	
C13A	0.32281 (12)	0.28819 (8)	0.28830 (6)	0.0162 (2)	
H13A	0.2728	0.2533	0.2594	0.019*	
H13B	0.3628	0.3159	0.2632	0.019*	
C14A	0.47177 (12)	0.15555 (7)	0.29804 (7)	0.0163 (2)	
C15A	0.54633 (13)	0.10433 (9)	0.32842 (7)	0.0217 (3)	
H15A	0.5753	0.1102	0.3723	0.026*	
C16A	0.57737 (14)	0.04462 (9)	0.29348 (8)	0.0243 (3)	
H16A	0.6276	0.0110	0.3138	0.029*	
C17A	0.53324 (14)	0.03528 (8)	0.22813 (8)	0.0230 (3)	
H17A	0.5542	−0.0046	0.2046	0.028*	
C18A	0.45801 (14)	0.08520 (8)	0.19785 (7)	0.0219 (3)	
H18A	0.4278	0.0784	0.1541	0.026*	
C19A	0.42734 (13)	0.14551 (8)	0.23259 (7)	0.0184 (2)	
H19A	0.3771	0.1791	0.2120	0.022*	
C20A	0.55895 (12)	0.30004 (8)	0.37121 (7)	0.0171 (2)	
C21A	0.56048 (13)	0.36082 (8)	0.41418 (7)	0.0208 (3)	
H21A	0.5007	0.3686	0.4332	0.025*	
C22A	0.65110 (15)	0.40982 (9)	0.42867 (8)	0.0267 (3)	
H22A	0.6512	0.4509	0.4567	0.032*	
C23A	0.74138 (14)	0.39751 (10)	0.40137 (9)	0.0288 (3)	
H23A	0.8021	0.4302	0.4112	0.035*	
C24A	0.74112 (14)	0.33647 (10)	0.35934 (9)	0.0271 (3)	
H24A	0.8021	0.3279	0.3415	0.033*	
C25A	0.64965 (13)	0.28783 (9)	0.34373 (8)	0.0216 (3)	
H25A	0.6492	0.2474	0.3151	0.026*	
C26A	0.13545 (13)	0.13752 (9)	0.67551 (7)	0.0226 (3)	
H26A	0.2071	0.1510	0.6666	0.027*	
C27A	0.1157 (2)	0.18750 (15)	0.73067 (11)	0.0520 (5)	
H27A	0.1225	0.2381	0.7213	0.078*	
H27B	0.1698	0.1780	0.7689	0.078*	
H27C	0.0424	0.1781	0.7368	0.078*	
C28A	0.13514 (19)	0.05692 (11)	0.68819 (11)	0.0395 (5)	
H28A	0.1436	0.0279	0.6509	0.059*	
H28B	0.0660	0.0437	0.6982	0.059*	
H28C	0.1956	0.0477	0.7235	0.059*	
C29A	−0.16430 (12)	0.16323 (9)	0.53451 (8)	0.0219 (3)	
H29A	−0.1631	0.1786	0.5791	0.026*	
C30A	−0.17230 (18)	0.08025 (11)	0.52515 (13)	0.0427 (4)	
H30A	−0.1106	0.0593	0.5537	0.064*	
H30B	−0.1707	0.0654	0.4818	0.064*	
H30C	−0.2407	0.0632	0.5340	0.064*	
C31A	−0.25866 (18)	0.19967 (14)	0.49048 (12)	0.0440 (4)	
H31A	−0.2528	0.2521	0.4996	0.066*	
H31B	−0.3283	0.1818	0.4969	0.066*	
H31C	−0.2549	0.1883	0.4469	0.066*	
C32A	0.04283 (14)	0.35253 (8)	0.59024 (8)	0.0223 (3)	
H32A	0.0905	0.3536	0.5596	0.027*	
C33A	−0.07353 (16)	0.37404 (10)	0.55800 (10)	0.0302 (4)	
H33A	−0.0996	0.3430	0.5197	0.045*	
H33B	−0.0728	0.4246	0.5474	0.045*	
H33C	−0.1221	0.3683	0.5865	0.045*	
C34A	0.09399 (19)	0.40151 (10)	0.64906 (10)	0.0348 (4)	
H34A	0.1656	0.3833	0.6688	0.052*	
H34B	0.0466	0.4014	0.6787	0.052*	
H34C	0.1023	0.4509	0.6370	0.052*	
C35A	0.05387 (13)	0.25422 (8)	0.38600 (7)	0.0198 (3)	
C36A	0.09066 (13)	0.38836 (8)	0.44165 (7)	0.0186 (2)	
C37A	0.30942 (13)	0.36302 (8)	0.48535 (7)	0.0194 (2)	
C38A	0.44768 (12)	0.23894 (8)	0.50448 (7)	0.0181 (2)	
C39A	0.40018 (13)	0.09858 (9)	0.44396 (7)	0.0209 (3)	
C40A	0.22252 (12)	0.15734 (8)	0.36194 (7)	0.0189 (2)	
C41A	0.10377 (13)	0.12908 (8)	0.45433 (7)	0.0207 (3)	
C42A	0.27394 (13)	0.11760 (9)	0.55825 (7)	0.0208 (3)	
C43A	0.28449 (13)	0.26821 (9)	0.58020 (7)	0.0211 (3)	
Ru1B	0.339824 (9)	0.737543 (6)	0.020451 (5)	0.01278 (2)	
Ru2B	0.554997 (9)	0.807238 (6)	0.065368 (5)	0.01303 (2)	
Ru3B	0.409502 (9)	0.797940 (6)	0.148373 (5)	0.01333 (2)	
As1B	0.354067 (12)	0.725193 (8)	−0.090391 (7)	0.01419 (2)	
As2B	0.617117 (12)	0.757903 (8)	−0.027337 (7)	0.01422 (2)	
P1B	0.24434 (3)	0.79901 (2)	0.179505 (17)	0.01526 (6)	
O1B	0.48228 (10)	0.60037 (6)	0.04909 (6)	0.0230 (2)	
O2B	0.12319 (10)	0.65451 (7)	0.01519 (7)	0.0297 (3)	
O3B	0.19725 (10)	0.87497 (6)	−0.00891 (6)	0.0250 (2)	
O4B	0.43815 (10)	0.92849 (6)	−0.01740 (6)	0.0259 (2)	
O5B	0.72668 (10)	0.92191 (7)	0.13537 (6)	0.0285 (2)	
O6B	0.67389 (10)	0.68923 (6)	0.15124 (6)	0.0234 (2)	
O7B	0.44806 (12)	0.63705 (7)	0.18197 (6)	0.0296 (3)	
O8B	0.58619 (11)	0.84901 (8)	0.26636 (6)	0.0324 (3)	
O9B	0.35312 (11)	0.95402 (6)	0.10515 (6)	0.0254 (2)	
O10B	0.24208 (10)	0.83223 (7)	0.24973 (6)	0.0260 (2)	
O11B	0.17395 (9)	0.72522 (6)	0.17894 (6)	0.0217 (2)	
O12B	0.15674 (9)	0.84427 (6)	0.13157 (5)	0.0203 (2)	
C1B	0.33933 (12)	0.62820 (7)	−0.13510 (7)	0.0163 (2)	
C2B	0.35030 (13)	0.62110 (8)	−0.19829 (7)	0.0201 (3)	
H2BA	0.3574	0.6630	−0.2200	0.024*	
C3B	0.35059 (14)	0.55150 (9)	−0.22884 (7)	0.0227 (3)	
H3BA	0.3578	0.5468	−0.2709	0.027*	
C4B	0.34000 (13)	0.48894 (9)	−0.19637 (8)	0.0227 (3)	
H4BA	0.3419	0.4423	−0.2165	0.027*	
C5B	0.32664 (12)	0.49591 (8)	−0.13416 (7)	0.0200 (3)	
H5BA	0.3180	0.4540	−0.1129	0.024*	
C6B	0.32616 (12)	0.56564 (8)	−0.10337 (7)	0.0177 (2)	
H6BA	0.3170	0.5702	−0.0616	0.021*	
C7B	0.25050 (12)	0.78285 (8)	−0.14899 (7)	0.0169 (2)	
C8B	0.27299 (15)	0.85673 (9)	−0.15500 (9)	0.0279 (3)	
H8BA	0.3416	0.8778	−0.1343	0.033*	
C9B	0.19267 (16)	0.89929 (9)	−0.19204 (9)	0.0317 (4)	
H9BA	0.2081	0.9487	−0.1961	0.038*	
C10B	0.09035 (15)	0.86873 (9)	−0.22268 (8)	0.0272 (3)	
H10B	0.0372	0.8972	−0.2477	0.033*	
C11B	0.06720 (13)	0.79510 (9)	−0.21592 (8)	0.0235 (3)	
H11B	−0.0020	0.7745	−0.2360	0.028*	
C12B	0.14688 (12)	0.75218 (8)	−0.17930 (7)	0.0197 (3)	
H12B	0.1310	0.7029	−0.1750	0.024*	
C13B	0.49808 (12)	0.75811 (8)	−0.10505 (7)	0.0188 (2)	
H13C	0.5175	0.7258	−0.1382	0.023*	
H13D	0.4912	0.8076	−0.1199	0.023*	
C14B	0.67501 (13)	0.65916 (8)	−0.02879 (7)	0.0190 (3)	
C15B	0.77084 (15)	0.64590 (9)	0.01763 (8)	0.0255 (3)	
H15B	0.7999	0.6823	0.0495	0.031*	
C16B	0.82291 (18)	0.57824 (10)	0.01631 (10)	0.0337 (4)	
H16B	0.8874	0.5696	0.0468	0.040*	
C17B	0.77798 (18)	0.52375 (10)	−0.03081 (11)	0.0370 (5)	
H17B	0.8129	0.4787	−0.0320	0.044*	
C18B	0.68180 (17)	0.53615 (10)	−0.07589 (11)	0.0357 (4)	
H18B	0.6513	0.4990	−0.1067	0.043*	
C19B	0.63018 (14)	0.60414 (9)	−0.07547 (9)	0.0265 (3)	
H19B	0.5660	0.6126	−0.1063	0.032*	
C20B	0.73519 (12)	0.81087 (8)	−0.05346 (7)	0.0173 (2)	
C21B	0.80847 (14)	0.77348 (9)	−0.08396 (8)	0.0247 (3)	
H21B	0.7994	0.7229	−0.0937	0.030*	
C22B	0.89520 (16)	0.81160 (10)	−0.09990 (10)	0.0316 (4)	
H22B	0.9443	0.7864	−0.1201	0.038*	
C23B	0.90882 (15)	0.88701 (10)	−0.08582 (10)	0.0307 (4)	
H23B	0.9672	0.9124	−0.0963	0.037*	
C24B	0.83519 (14)	0.92459 (9)	−0.05607 (9)	0.0267 (3)	
H24B	0.8437	0.9753	−0.0472	0.032*	
C25B	0.74863 (12)	0.88669 (8)	−0.03947 (8)	0.0208 (3)	
H25B	0.6999	0.9120	−0.0191	0.025*	
C26B	0.31227 (16)	0.89327 (10)	0.28401 (9)	0.0305 (4)	
H26B	0.3632	0.9091	0.2583	0.037*	
C27B	0.2382 (2)	0.95604 (15)	0.29488 (11)	0.0520 (5)	
H27D	0.1988	0.9722	0.2544	0.078*	
H27E	0.2828	0.9962	0.3190	0.078*	
H27F	0.1858	0.9397	0.3182	0.078*	
C28B	0.37867 (18)	0.86573 (14)	0.34603 (9)	0.0392 (4)	
H28D	0.4231	0.8251	0.3369	0.059*	
H28E	0.3287	0.8498	0.3710	0.059*	
H28F	0.4263	0.9048	0.3696	0.059*	
C29B	0.20585 (15)	0.66959 (10)	0.22553 (10)	0.0293 (4)	
H29B	0.2850	0.6757	0.2464	0.035*	
C30B	0.18501 (18)	0.59571 (11)	0.18843 (13)	0.0427 (4)	
H30D	0.2279	0.5930	0.1564	0.064*	
H30E	0.1074	0.5900	0.1682	0.064*	
H30F	0.2065	0.5571	0.2170	0.064*	
C31B	0.13696 (18)	0.67900 (14)	0.27457 (12)	0.0440 (4)	
H31D	0.1547	0.7259	0.2980	0.066*	
H31E	0.1527	0.6403	0.3035	0.066*	
H31F	0.0594	0.6769	0.2535	0.066*	
C32B	0.04202 (12)	0.85458 (9)	0.13787 (8)	0.0227 (3)	
H32B	0.0375	0.8471	0.1819	0.027*	
C33B	−0.03334 (15)	0.79981 (11)	0.09303 (11)	0.0365 (4)	
H33D	−0.0127	0.7507	0.1045	0.055*	
H33E	−0.0266	0.8058	0.0501	0.055*	
H33F	−0.1089	0.8078	0.0958	0.055*	
C34B	0.01410 (15)	0.93339 (10)	0.12292 (11)	0.0319 (4)	
H34D	0.0661	0.9663	0.1518	0.048*	
H34E	−0.0599	0.9433	0.1278	0.048*	
H34F	0.0184	0.9407	0.0797	0.048*	
C35B	0.43313 (12)	0.65362 (8)	0.04108 (7)	0.0171 (2)	
C36B	0.20505 (12)	0.68588 (8)	0.01746 (7)	0.0191 (2)	
C37B	0.25441 (12)	0.82674 (8)	0.00457 (7)	0.0179 (2)	
C38B	0.47542 (12)	0.88203 (8)	0.01420 (7)	0.0184 (2)	
C39B	0.66404 (12)	0.87716 (8)	0.10905 (7)	0.0182 (2)	
C40B	0.62378 (12)	0.73118 (8)	0.11932 (7)	0.0178 (2)	
C41B	0.43382 (13)	0.69593 (8)	0.16652 (7)	0.0203 (3)	
C42B	0.51685 (13)	0.83132 (9)	0.22284 (7)	0.0213 (3)	
C43B	0.37617 (12)	0.89545 (8)	0.11856 (7)	0.0188 (2)	

### Atomic displacement parameters (Å^2^)

**Table:**

	*U*^11^	*U*^22^	*U*^33^	*U*^12^	*U*^13^	*U*^23^
Ru1A	0.01498 (4)	0.01348 (4)	0.01328 (4)	0.00320 (3)	0.00426 (3)	0.00231 (3)
Ru2A	0.01373 (4)	0.01421 (4)	0.01253 (4)	0.00283 (3)	0.00234 (3)	0.00207 (3)
Ru3A	0.01465 (4)	0.01462 (4)	0.01440 (4)	0.00116 (3)	0.00371 (4)	0.00334 (3)
As1A	0.01435 (6)	0.01388 (6)	0.01247 (6)	0.00331 (4)	0.00323 (4)	0.00196 (4)
As2A	0.01417 (6)	0.01534 (6)	0.01208 (6)	0.00351 (4)	0.00263 (4)	0.00179 (4)
P1A	0.01446 (14)	0.01624 (15)	0.01473 (14)	0.00120 (11)	0.00285 (12)	0.00451 (11)
O1A	0.0223 (5)	0.0260 (6)	0.0318 (6)	0.0010 (4)	0.0012 (5)	0.0003 (5)
O2A	0.0316 (6)	0.0217 (5)	0.0361 (7)	0.0102 (5)	0.0170 (5)	0.0054 (5)
O3A	0.0284 (6)	0.0238 (5)	0.0243 (6)	0.0000 (4)	−0.0017 (5)	0.0002 (4)
O4A	0.0218 (5)	0.0287 (6)	0.0209 (5)	0.0021 (4)	−0.0001 (4)	−0.0016 (4)
O5A	0.0432 (8)	0.0236 (6)	0.0329 (7)	0.0138 (5)	0.0039 (6)	0.0060 (5)
O6A	0.0214 (5)	0.0308 (6)	0.0246 (6)	0.0032 (4)	−0.0008 (4)	−0.0047 (5)
O7A	0.0334 (6)	0.0240 (6)	0.0284 (6)	−0.0051 (5)	0.0038 (5)	−0.0012 (5)
O8A	0.0347 (7)	0.0281 (6)	0.0285 (6)	0.0116 (5)	0.0059 (5)	0.0110 (5)
O9A	0.0289 (6)	0.0367 (7)	0.0241 (6)	−0.0104 (5)	0.0054 (5)	−0.0039 (5)
O10A	0.0167 (4)	0.0221 (5)	0.0202 (5)	0.0008 (4)	0.0026 (4)	0.0104 (4)
O11A	0.0148 (4)	0.0246 (5)	0.0178 (5)	0.0010 (4)	0.0020 (4)	0.0049 (4)
O12A	0.0248 (5)	0.0181 (5)	0.0181 (5)	0.0012 (4)	0.0084 (4)	0.0034 (4)
C1A	0.0148 (5)	0.0143 (5)	0.0159 (6)	0.0017 (4)	0.0011 (4)	0.0023 (4)
C2A	0.0174 (6)	0.0307 (8)	0.0205 (7)	0.0070 (5)	0.0032 (5)	0.0026 (6)
C3A	0.0183 (7)	0.0320 (8)	0.0293 (8)	0.0089 (6)	0.0017 (6)	0.0040 (6)
C4A	0.0165 (6)	0.0227 (7)	0.0267 (7)	0.0005 (5)	−0.0026 (5)	0.0089 (6)
C5A	0.0242 (7)	0.0336 (8)	0.0186 (7)	0.0049 (6)	0.0014 (6)	0.0080 (6)
C6A	0.0221 (7)	0.0304 (8)	0.0178 (6)	0.0097 (6)	0.0034 (5)	0.0050 (5)
C7A	0.0160 (5)	0.0167 (6)	0.0148 (5)	0.0018 (4)	0.0029 (4)	0.0031 (4)
C8A	0.0269 (7)	0.0210 (6)	0.0213 (7)	0.0017 (5)	0.0111 (6)	0.0025 (5)
C9A	0.0245 (7)	0.0235 (7)	0.0267 (7)	−0.0003 (5)	0.0113 (6)	0.0063 (6)
C10A	0.0216 (7)	0.0209 (6)	0.0230 (7)	−0.0033 (5)	0.0022 (5)	0.0034 (5)
C11A	0.0265 (7)	0.0208 (7)	0.0216 (7)	−0.0037 (5)	0.0058 (6)	−0.0023 (5)
C12A	0.0214 (6)	0.0192 (6)	0.0186 (6)	−0.0009 (5)	0.0064 (5)	0.0003 (5)
C13A	0.0175 (6)	0.0171 (6)	0.0138 (5)	0.0054 (4)	0.0032 (4)	0.0018 (4)
C14A	0.0181 (6)	0.0155 (5)	0.0159 (6)	0.0027 (4)	0.0051 (5)	0.0016 (4)
C15A	0.0234 (7)	0.0232 (7)	0.0178 (6)	0.0089 (5)	0.0031 (5)	0.0012 (5)
C16A	0.0272 (7)	0.0216 (7)	0.0243 (7)	0.0100 (6)	0.0057 (6)	0.0032 (5)
C17A	0.0314 (8)	0.0162 (6)	0.0230 (7)	0.0043 (5)	0.0095 (6)	0.0011 (5)
C18A	0.0324 (8)	0.0161 (6)	0.0172 (6)	0.0016 (5)	0.0056 (6)	0.0005 (5)
C19A	0.0240 (7)	0.0151 (6)	0.0158 (6)	0.0023 (5)	0.0033 (5)	0.0020 (4)
C20A	0.0158 (6)	0.0189 (6)	0.0159 (6)	0.0016 (4)	0.0013 (5)	0.0044 (5)
C21A	0.0211 (6)	0.0218 (6)	0.0188 (6)	0.0008 (5)	0.0029 (5)	0.0016 (5)
C22A	0.0288 (8)	0.0247 (7)	0.0233 (7)	−0.0051 (6)	−0.0007 (6)	0.0015 (6)
C23A	0.0226 (7)	0.0323 (8)	0.0292 (8)	−0.0071 (6)	−0.0008 (6)	0.0108 (7)
C24A	0.0178 (6)	0.0352 (8)	0.0294 (8)	0.0010 (6)	0.0050 (6)	0.0116 (7)
C25A	0.0185 (6)	0.0260 (7)	0.0215 (7)	0.0035 (5)	0.0059 (5)	0.0059 (5)
C26A	0.0204 (6)	0.0299 (7)	0.0183 (6)	0.0050 (5)	0.0036 (5)	0.0106 (5)
C27A	0.0609 (11)	0.0565 (10)	0.0322 (7)	0.0290 (9)	−0.0004 (7)	−0.0087 (7)
C28A	0.0400 (10)	0.0346 (10)	0.0419 (11)	0.0058 (8)	0.0000 (9)	0.0227 (8)
C29A	0.0155 (6)	0.0260 (7)	0.0242 (7)	−0.0007 (5)	0.0044 (5)	0.0035 (5)
C30A	0.0312 (7)	0.0245 (6)	0.0691 (11)	−0.0042 (5)	0.0036 (7)	0.0074 (6)
C31A	0.0319 (7)	0.0525 (9)	0.0538 (9)	0.0109 (6)	0.0167 (7)	0.0258 (8)
C32A	0.0283 (7)	0.0176 (6)	0.0243 (7)	−0.0001 (5)	0.0124 (6)	0.0028 (5)
C33A	0.0337 (9)	0.0229 (7)	0.0383 (9)	0.0087 (6)	0.0150 (7)	0.0102 (7)
C34A	0.0459 (11)	0.0261 (8)	0.0351 (9)	−0.0104 (7)	0.0176 (8)	−0.0073 (7)
C35A	0.0209 (6)	0.0179 (6)	0.0214 (6)	0.0045 (5)	0.0061 (5)	0.0019 (5)
C36A	0.0214 (6)	0.0178 (6)	0.0185 (6)	0.0023 (5)	0.0075 (5)	0.0039 (5)
C37A	0.0212 (6)	0.0198 (6)	0.0174 (6)	0.0044 (5)	0.0044 (5)	0.0041 (5)
C38A	0.0183 (6)	0.0195 (6)	0.0167 (6)	0.0036 (5)	0.0044 (5)	0.0023 (5)
C39A	0.0220 (7)	0.0221 (6)	0.0180 (6)	0.0051 (5)	0.0031 (5)	0.0026 (5)
C40A	0.0165 (6)	0.0206 (6)	0.0193 (6)	0.0043 (5)	0.0030 (5)	0.0022 (5)
C41A	0.0234 (7)	0.0190 (6)	0.0204 (6)	0.0017 (5)	0.0059 (5)	0.0038 (5)
C42A	0.0213 (6)	0.0222 (6)	0.0198 (6)	0.0021 (5)	0.0057 (5)	0.0049 (5)
C43A	0.0188 (6)	0.0249 (7)	0.0207 (6)	−0.0016 (5)	0.0064 (5)	0.0033 (5)
Ru1B	0.01209 (4)	0.01290 (4)	0.01270 (4)	−0.00062 (3)	0.00130 (3)	0.00173 (3)
Ru2B	0.01213 (4)	0.01236 (4)	0.01426 (4)	−0.00093 (3)	0.00238 (3)	0.00084 (3)
Ru3B	0.01277 (4)	0.01367 (4)	0.01320 (4)	0.00093 (3)	0.00229 (3)	0.00055 (3)
As1B	0.01590 (6)	0.01336 (6)	0.01246 (6)	−0.00087 (4)	0.00123 (5)	0.00213 (4)
As2B	0.01477 (6)	0.01318 (6)	0.01513 (6)	−0.00051 (4)	0.00402 (5)	0.00199 (4)
P1B	0.01455 (14)	0.01611 (15)	0.01531 (15)	0.00140 (11)	0.00363 (12)	0.00172 (11)
O1B	0.0216 (5)	0.0187 (5)	0.0261 (5)	0.0022 (4)	−0.0001 (4)	0.0031 (4)
O2B	0.0191 (5)	0.0244 (6)	0.0455 (8)	−0.0045 (4)	0.0067 (5)	0.0045 (5)
O3B	0.0235 (5)	0.0208 (5)	0.0285 (6)	0.0046 (4)	0.0012 (4)	0.0027 (4)
O4B	0.0250 (5)	0.0199 (5)	0.0303 (6)	−0.0011 (4)	−0.0002 (5)	0.0078 (4)
O5B	0.0245 (6)	0.0249 (6)	0.0321 (6)	−0.0076 (4)	0.0004 (5)	−0.0040 (5)
O6B	0.0236 (5)	0.0220 (5)	0.0239 (5)	0.0024 (4)	0.0029 (4)	0.0062 (4)
O7B	0.0389 (7)	0.0230 (5)	0.0335 (6)	0.0101 (5)	0.0191 (6)	0.0111 (5)
O8B	0.0233 (6)	0.0414 (7)	0.0266 (6)	0.0002 (5)	−0.0048 (5)	−0.0052 (5)
O9B	0.0295 (6)	0.0161 (5)	0.0284 (6)	0.0023 (4)	0.0023 (5)	0.0004 (4)
O10B	0.0249 (5)	0.0348 (6)	0.0191 (5)	0.0018 (5)	0.0086 (4)	−0.0049 (4)
O11B	0.0185 (5)	0.0195 (5)	0.0275 (5)	−0.0011 (4)	0.0050 (4)	0.0069 (4)
O12B	0.0142 (4)	0.0243 (5)	0.0238 (5)	0.0038 (4)	0.0053 (4)	0.0094 (4)
C1B	0.0165 (6)	0.0157 (5)	0.0157 (6)	0.0001 (4)	0.0018 (5)	0.0012 (4)
C2B	0.0240 (7)	0.0197 (6)	0.0164 (6)	0.0009 (5)	0.0038 (5)	0.0020 (5)
C3B	0.0257 (7)	0.0237 (7)	0.0180 (6)	0.0050 (5)	0.0044 (5)	−0.0020 (5)
C4B	0.0213 (7)	0.0189 (6)	0.0259 (7)	0.0053 (5)	0.0020 (6)	−0.0023 (5)
C5B	0.0190 (6)	0.0154 (6)	0.0237 (7)	0.0024 (5)	0.0007 (5)	0.0026 (5)
C6B	0.0174 (6)	0.0169 (6)	0.0174 (6)	0.0002 (5)	0.0009 (5)	0.0025 (5)
C7B	0.0195 (6)	0.0164 (6)	0.0134 (5)	0.0003 (5)	0.0004 (5)	0.0031 (4)
C8B	0.0280 (8)	0.0193 (7)	0.0299 (8)	−0.0057 (6)	−0.0082 (6)	0.0070 (6)
C9B	0.0342 (9)	0.0175 (7)	0.0358 (9)	−0.0042 (6)	−0.0096 (7)	0.0093 (6)
C10B	0.0279 (8)	0.0227 (7)	0.0266 (8)	0.0023 (6)	−0.0048 (6)	0.0082 (6)
C11B	0.0196 (6)	0.0243 (7)	0.0242 (7)	−0.0015 (5)	−0.0007 (5)	0.0065 (6)
C12B	0.0195 (6)	0.0177 (6)	0.0207 (6)	−0.0017 (5)	0.0015 (5)	0.0051 (5)
C13B	0.0197 (6)	0.0210 (6)	0.0154 (6)	−0.0034 (5)	0.0029 (5)	0.0036 (5)
C14B	0.0218 (6)	0.0145 (6)	0.0236 (7)	0.0017 (5)	0.0105 (5)	0.0035 (5)
C15B	0.0304 (8)	0.0229 (7)	0.0250 (7)	0.0069 (6)	0.0083 (6)	0.0074 (6)
C16B	0.0402 (10)	0.0287 (8)	0.0374 (10)	0.0149 (7)	0.0156 (8)	0.0155 (7)
C17B	0.0456 (11)	0.0184 (7)	0.0571 (13)	0.0101 (7)	0.0303 (10)	0.0108 (7)
C18B	0.0372 (10)	0.0176 (7)	0.0583 (13)	−0.0025 (6)	0.0253 (9)	−0.0054 (7)
C19B	0.0231 (7)	0.0199 (7)	0.0380 (9)	−0.0025 (5)	0.0119 (7)	−0.0045 (6)
C20B	0.0158 (6)	0.0187 (6)	0.0181 (6)	−0.0006 (4)	0.0041 (5)	0.0055 (5)
C21B	0.0277 (7)	0.0213 (7)	0.0297 (8)	0.0011 (6)	0.0152 (6)	0.0042 (6)
C22B	0.0305 (8)	0.0283 (8)	0.0443 (10)	0.0042 (6)	0.0237 (8)	0.0099 (7)
C23B	0.0230 (7)	0.0292 (8)	0.0460 (10)	0.0018 (6)	0.0166 (7)	0.0163 (7)
C24B	0.0223 (7)	0.0196 (7)	0.0406 (9)	−0.0012 (5)	0.0099 (7)	0.0102 (6)
C25B	0.0184 (6)	0.0171 (6)	0.0285 (7)	0.0006 (5)	0.0071 (5)	0.0069 (5)
C26B	0.0317 (9)	0.0329 (9)	0.0235 (8)	0.0074 (7)	0.0020 (6)	−0.0100 (6)
C27B	0.0609 (11)	0.0565 (10)	0.0322 (7)	0.0290 (9)	−0.0004 (7)	−0.0087 (7)
C28B	0.0327 (10)	0.0588 (13)	0.0236 (8)	0.0047 (9)	0.0029 (7)	−0.0031 (8)
C29B	0.0235 (7)	0.0282 (8)	0.0414 (10)	0.0070 (6)	0.0139 (7)	0.0184 (7)
C30B	0.0312 (7)	0.0245 (6)	0.0691 (11)	−0.0042 (5)	0.0036 (7)	0.0074 (6)
C31B	0.0319 (7)	0.0525 (9)	0.0538 (9)	0.0109 (6)	0.0167 (7)	0.0258 (8)
C32B	0.0143 (6)	0.0250 (7)	0.0309 (8)	0.0024 (5)	0.0075 (5)	0.0096 (6)
C33B	0.0189 (7)	0.0316 (9)	0.0541 (12)	0.0000 (6)	−0.0023 (8)	0.0058 (8)
C34B	0.0204 (7)	0.0247 (8)	0.0514 (11)	0.0059 (6)	0.0074 (7)	0.0113 (7)
C35B	0.0159 (6)	0.0191 (6)	0.0153 (6)	−0.0015 (5)	0.0011 (5)	0.0025 (4)
C36B	0.0184 (6)	0.0159 (6)	0.0221 (6)	0.0010 (5)	0.0026 (5)	0.0026 (5)
C37B	0.0170 (6)	0.0174 (6)	0.0178 (6)	−0.0021 (5)	0.0011 (5)	0.0009 (5)
C38B	0.0156 (6)	0.0165 (6)	0.0220 (6)	−0.0019 (4)	0.0022 (5)	0.0006 (5)
C39B	0.0168 (6)	0.0179 (6)	0.0192 (6)	0.0001 (5)	0.0028 (5)	0.0008 (5)
C40B	0.0174 (6)	0.0172 (6)	0.0184 (6)	−0.0023 (5)	0.0038 (5)	0.0001 (5)
C41B	0.0205 (6)	0.0221 (6)	0.0208 (6)	0.0043 (5)	0.0094 (5)	0.0042 (5)
C42B	0.0197 (6)	0.0231 (7)	0.0203 (6)	0.0023 (5)	0.0034 (5)	0.0001 (5)
C43B	0.0190 (6)	0.0186 (6)	0.0173 (6)	−0.0003 (5)	0.0016 (5)	−0.0005 (5)

### Geometric parameters (Å, °)

**Table:**

Ru1A—C36A	1.8731 (14)	Ru1B—C36B	1.8914 (15)
Ru1A—C37A	1.9246 (16)	Ru1B—C35B	1.9292 (14)
Ru1A—C35A	1.9386 (16)	Ru1B—C37B	1.9445 (14)
Ru1A—As1A	2.45955 (17)	Ru1B—As1B	2.42970 (18)
Ru1A—Ru2A	2.87808 (16)	Ru1B—Ru3B	2.83973 (16)
Ru1A—Ru3A	2.90929 (15)	Ru1B—Ru2B	2.86845 (16)
Ru2A—C39A	1.8945 (15)	Ru2B—C39B	1.8876 (15)
Ru2A—C40A	1.9225 (15)	Ru2B—C38B	1.9315 (15)
Ru2A—C38A	1.9343 (15)	Ru2B—C40B	1.9340 (15)
Ru2A—As2A	2.41356 (17)	Ru2B—As2B	2.42226 (18)
Ru2A—Ru3A	2.81062 (15)	Ru2B—Ru3B	2.81996 (15)
Ru3A—C42A	1.8891 (15)	Ru3B—C42B	1.8955 (16)
Ru3A—C43A	1.9298 (16)	Ru3B—C41B	1.9287 (15)
Ru3A—C41A	1.9491 (16)	Ru3B—C43B	1.9359 (15)
Ru3A—P1A	2.2926 (4)	Ru3B—P1B	2.2877 (4)
As1A—C7A	1.9494 (14)	As1B—C7B	1.9404 (14)
As1A—C1A	1.9532 (13)	As1B—C1B	1.9426 (14)
As1A—C13A	1.9638 (13)	As1B—C13B	1.9711 (14)
As2A—C14A	1.9363 (14)	As2B—C14B	1.9369 (14)
As2A—C20A	1.9426 (14)	As2B—C20B	1.9470 (13)
As2A—C13A	1.9626 (13)	As2B—C13B	1.9583 (15)
P1A—O11A	1.5839 (11)	P1B—O12B	1.5860 (11)
P1A—O10A	1.5935 (10)	P1B—O11B	1.5909 (11)
P1A—O12A	1.6003 (11)	P1B—O10B	1.5997 (12)
O1A—C35A	1.142 (2)	O1B—C35B	1.1464 (18)
O2A—C36A	1.1505 (18)	O2B—C36B	1.1469 (18)
O3A—C37A	1.149 (2)	O3B—C37B	1.1384 (18)
O4A—C38A	1.1437 (19)	O4B—C38B	1.1486 (18)
O5A—C39A	1.1441 (19)	O5B—C39B	1.1470 (18)
O6A—C40A	1.1489 (19)	O6B—C40B	1.1452 (18)
O7A—C41A	1.136 (2)	O7B—C41B	1.1426 (18)
O8A—C42A	1.1410 (19)	O8B—C42B	1.143 (2)
O9A—C43A	1.145 (2)	O9B—C43B	1.1406 (18)
O10A—C26A	1.4584 (19)	O10B—C26B	1.456 (2)
O11A—C29A	1.4697 (18)	O11B—C29B	1.4596 (19)
O12A—C32A	1.4595 (18)	O12B—C32B	1.4658 (18)
C1A—C6A	1.390 (2)	C1B—C6B	1.3887 (19)
C1A—C2A	1.391 (2)	C1B—C2B	1.396 (2)
C2A—C3A	1.396 (2)	C2B—C3B	1.390 (2)
C2A—H2AA	0.9300	C2B—H2BA	0.9300
C3A—C4A	1.383 (2)	C3B—C4B	1.391 (2)
C3A—H3AA	0.9300	C3B—H3BA	0.9300
C4A—C5A	1.379 (2)	C4B—C5B	1.385 (2)
C4A—H4AA	0.9300	C4B—H4BA	0.9300
C5A—C6A	1.397 (2)	C5B—C6B	1.395 (2)
C5A—H5AA	0.9300	C5B—H5BA	0.9300
C6A—H6AA	0.9300	C6B—H6BA	0.9300
C7A—C8A	1.392 (2)	C7B—C8B	1.389 (2)
C7A—C12A	1.395 (2)	C7B—C12B	1.392 (2)
C8A—C9A	1.392 (2)	C8B—C9B	1.393 (2)
C8A—H8AA	0.9300	C8B—H8BA	0.9300
C9A—C10A	1.383 (2)	C9B—C10B	1.380 (2)
C9A—H9AA	0.9300	C9B—H9BA	0.9300
C10A—C11A	1.391 (2)	C10B—C11B	1.389 (2)
C10A—H10A	0.9300	C10B—H10B	0.9300
C11A—C12A	1.391 (2)	C11B—C12B	1.389 (2)
C11A—H11A	0.9300	C11B—H11B	0.9300
C12A—H12A	0.9300	C12B—H12B	0.9300
C13A—H13A	0.9700	C13B—H13C	0.9700
C13A—H13B	0.9700	C13B—H13D	0.9700
C14A—C19A	1.391 (2)	C14B—C19B	1.390 (2)
C14A—C15A	1.397 (2)	C14B—C15B	1.396 (2)
C15A—C16A	1.388 (2)	C15B—C16B	1.393 (2)
C15A—H15A	0.9300	C15B—H15B	0.9300
C16A—C17A	1.387 (2)	C16B—C17B	1.389 (3)
C16A—H16A	0.9300	C16B—H16B	0.9300
C17A—C18A	1.385 (2)	C17B—C18B	1.380 (3)
C17A—H17A	0.9300	C17B—H17B	0.9300
C18A—C19A	1.393 (2)	C18B—C19B	1.394 (2)
C18A—H18A	0.9300	C18B—H18B	0.9300
C19A—H19A	0.9300	C19B—H19B	0.9300
C20A—C21A	1.394 (2)	C20B—C21B	1.392 (2)
C20A—C25A	1.394 (2)	C20B—C25B	1.393 (2)
C21A—C22A	1.391 (2)	C21B—C22B	1.390 (2)
C21A—H21A	0.9300	C21B—H21B	0.9300
C22A—C23A	1.387 (3)	C22B—C23B	1.386 (3)
C22A—H22A	0.9300	C22B—H22B	0.9300
C23A—C24A	1.388 (3)	C23B—C24B	1.387 (2)
C23A—H23A	0.9300	C23B—H23B	0.9300
C24A—C25A	1.395 (2)	C24B—C25B	1.393 (2)
C24A—H24A	0.9300	C24B—H24B	0.9300
C25A—H25A	0.9300	C25B—H25B	0.9300
C26A—C28A	1.506 (2)	C26B—C27B	1.507 (3)
C26A—C27A	1.516 (3)	C26B—C28B	1.514 (3)
C26A—H26A	0.9800	C26B—H26B	0.9800
C27A—H27A	0.9600	C27B—H27D	0.9600
C27A—H27B	0.9600	C27B—H27E	0.9600
C27A—H27C	0.9600	C27B—H27F	0.9600
C28A—H28A	0.9600	C28B—H28D	0.9600
C28A—H28B	0.9600	C28B—H28E	0.9600
C28A—H28C	0.9600	C28B—H28F	0.9600
C29A—C31A	1.509 (3)	C29B—C31B	1.503 (3)
C29A—C30A	1.509 (3)	C29B—C30B	1.509 (3)
C29A—H29A	0.9800	C29B—H29B	0.9800
C30A—H30A	0.9600	C30B—H30D	0.9600
C30A—H30B	0.9600	C30B—H30E	0.9600
C30A—H30C	0.9600	C30B—H30F	0.9600
C31A—H31A	0.9600	C31B—H31D	0.9600
C31A—H31B	0.9600	C31B—H31E	0.9600
C31A—H31C	0.9600	C31B—H31F	0.9600
C32A—C33A	1.511 (3)	C32B—C33B	1.499 (3)
C32A—C34A	1.511 (3)	C32B—C34B	1.512 (2)
C32A—H32A	0.9800	C32B—H32B	0.9800
C33A—H33A	0.9600	C33B—H33D	0.9600
C33A—H33B	0.9600	C33B—H33E	0.9600
C33A—H33C	0.9600	C33B—H33F	0.9600
C34A—H34A	0.9600	C34B—H34D	0.9600
C34A—H34B	0.9600	C34B—H34E	0.9600
C34A—H34C	0.9600	C34B—H34F	0.9600
			
C36A—Ru1A—C37A	90.69 (6)	C36B—Ru1B—C35B	95.01 (6)
C36A—Ru1A—C35A	87.60 (6)	C36B—Ru1B—C37B	88.31 (6)
C37A—Ru1A—C35A	167.69 (6)	C35B—Ru1B—C37B	175.52 (6)
C36A—Ru1A—As1A	98.87 (4)	C36B—Ru1B—As1B	102.23 (5)
C37A—Ru1A—As1A	93.49 (4)	C35B—Ru1B—As1B	91.41 (4)
C35A—Ru1A—As1A	98.82 (4)	C37B—Ru1B—As1B	90.82 (4)
C36A—Ru1A—Ru2A	168.90 (4)	C36B—Ru1B—Ru3B	105.09 (5)
C37A—Ru1A—Ru2A	83.29 (4)	C35B—Ru1B—Ru3B	90.48 (4)
C35A—Ru1A—Ru2A	96.28 (4)	C37B—Ru1B—Ru3B	85.74 (4)
As1A—Ru1A—Ru2A	90.831 (5)	As1B—Ru1B—Ru3B	152.336 (6)
C36A—Ru1A—Ru3A	112.69 (4)	C36B—Ru1B—Ru2B	162.49 (5)
C37A—Ru1A—Ru3A	89.28 (4)	C35B—Ru1B—Ru2B	78.46 (4)
C35A—Ru1A—Ru3A	80.16 (4)	C37B—Ru1B—Ru2B	97.50 (4)
As1A—Ru1A—Ru3A	148.290 (6)	As1B—Ru1B—Ru2B	94.217 (5)
Ru2A—Ru1A—Ru3A	58.107 (4)	Ru3B—Ru1B—Ru2B	59.208 (4)
C39A—Ru2A—C40A	92.15 (6)	C39B—Ru2B—C38B	91.23 (6)
C39A—Ru2A—C38A	93.44 (6)	C39B—Ru2B—C40B	91.03 (6)
C40A—Ru2A—C38A	173.78 (6)	C38B—Ru2B—C40B	175.25 (6)
C39A—Ru2A—As2A	100.95 (5)	C39B—Ru2B—As2B	106.85 (4)
C40A—Ru2A—As2A	85.40 (4)	C38B—Ru2B—As2B	89.22 (4)
C38A—Ru2A—As2A	96.18 (4)	C40B—Ru2B—As2B	94.12 (4)
C39A—Ru2A—Ru3A	100.87 (5)	C39B—Ru2B—Ru3B	103.35 (4)
C40A—Ru2A—Ru3A	92.12 (4)	C38B—Ru2B—Ru3B	96.62 (4)
C38A—Ru2A—Ru3A	84.17 (4)	C40B—Ru2B—Ru3B	78.79 (4)
As2A—Ru2A—Ru3A	158.123 (6)	As2B—Ru2B—Ru3B	149.091 (6)
C39A—Ru2A—Ru1A	159.78 (5)	C39B—Ru2B—Ru1B	158.69 (4)
C40A—Ru2A—Ru1A	79.49 (4)	C38B—Ru2B—Ru1B	78.77 (4)
C38A—Ru2A—Ru1A	94.34 (4)	C40B—Ru2B—Ru1B	97.72 (4)
As2A—Ru2A—Ru1A	96.728 (5)	As2B—Ru2B—Ru1B	91.922 (5)
Ru3A—Ru2A—Ru1A	61.503 (4)	Ru3B—Ru2B—Ru1B	59.889 (4)
C42A—Ru3A—C43A	91.93 (7)	C42B—Ru3B—C41B	92.30 (7)
C42A—Ru3A—C41A	90.06 (7)	C42B—Ru3B—C43B	94.97 (6)
C43A—Ru3A—C41A	177.11 (6)	C41B—Ru3B—C43B	172.34 (6)
C42A—Ru3A—P1A	99.67 (5)	C42B—Ru3B—P1B	104.57 (5)
C43A—Ru3A—P1A	90.64 (4)	C41B—Ru3B—P1B	92.60 (4)
C41A—Ru3A—P1A	86.96 (5)	C43B—Ru3B—P1B	87.93 (4)
C42A—Ru3A—Ru2A	89.59 (4)	C42B—Ru3B—Ru2B	94.91 (5)
C43A—Ru3A—Ru2A	96.93 (4)	C41B—Ru3B—Ru2B	97.33 (4)
C41A—Ru3A—Ru2A	85.17 (4)	C43B—Ru3B—Ru2B	79.65 (4)
P1A—Ru3A—Ru2A	167.852 (10)	P1B—Ru3B—Ru2B	157.752 (11)
C42A—Ru3A—Ru1A	149.34 (5)	C42B—Ru3B—Ru1B	154.24 (5)
C43A—Ru3A—Ru1A	86.14 (5)	C41B—Ru3B—Ru1B	82.95 (5)
C41A—Ru3A—Ru1A	93.21 (4)	C43B—Ru3B—Ru1B	89.44 (4)
P1A—Ru3A—Ru1A	110.938 (10)	P1B—Ru3B—Ru1B	100.939 (10)
Ru2A—Ru3A—Ru1A	60.390 (4)	Ru2B—Ru3B—Ru1B	60.904 (4)
C7A—As1A—C1A	96.02 (6)	C7B—As1B—C1B	102.58 (6)
C7A—As1A—C13A	103.29 (6)	C7B—As1B—C13B	101.61 (6)
C1A—As1A—C13A	100.05 (6)	C1B—As1B—C13B	100.11 (6)
C7A—As1A—Ru1A	117.13 (4)	C7B—As1B—Ru1B	115.57 (4)
C1A—As1A—Ru1A	120.27 (4)	C1B—As1B—Ru1B	119.61 (4)
C13A—As1A—Ru1A	116.55 (4)	C13B—As1B—Ru1B	114.69 (4)
C14A—As2A—C20A	102.99 (6)	C14B—As2B—C20B	99.33 (6)
C14A—As2A—C13A	101.98 (6)	C14B—As2B—C13B	104.39 (7)
C20A—As2A—C13A	102.67 (6)	C20B—As2B—C13B	100.91 (6)
C14A—As2A—Ru2A	114.16 (4)	C14B—As2B—Ru2B	119.98 (4)
C20A—As2A—Ru2A	122.44 (4)	C20B—As2B—Ru2B	118.26 (4)
C13A—As2A—Ru2A	110.20 (4)	C13B—As2B—Ru2B	111.40 (4)
O11A—P1A—O10A	100.18 (6)	O12B—P1B—O11B	100.20 (6)
O11A—P1A—O12A	107.60 (6)	O12B—P1B—O10B	106.00 (7)
O10A—P1A—O12A	98.03 (6)	O11B—P1B—O10B	98.48 (7)
O11A—P1A—Ru3A	110.48 (4)	O12B—P1B—Ru3B	109.20 (4)
O10A—P1A—Ru3A	117.33 (4)	O11B—P1B—Ru3B	121.52 (5)
O12A—P1A—Ru3A	120.75 (4)	O10B—P1B—Ru3B	118.99 (5)
C26A—O10A—P1A	123.23 (10)	C26B—O10B—P1B	124.91 (11)
C29A—O11A—P1A	125.30 (10)	C29B—O11B—P1B	123.45 (11)
C32A—O12A—P1A	123.41 (9)	C32B—O12B—P1B	122.81 (9)
C6A—C1A—C2A	119.26 (13)	C6B—C1B—C2B	119.84 (13)
C6A—C1A—As1A	121.58 (10)	C6B—C1B—As1B	120.55 (10)
C2A—C1A—As1A	118.78 (11)	C2B—C1B—As1B	119.44 (10)
C1A—C2A—C3A	119.99 (15)	C3B—C2B—C1B	120.03 (14)
C1A—C2A—H2AA	120.0	C3B—C2B—H2BA	120.0
C3A—C2A—H2AA	120.0	C1B—C2B—H2BA	120.0
C4A—C3A—C2A	120.36 (15)	C2B—C3B—C4B	119.92 (14)
C4A—C3A—H3AA	119.8	C2B—C3B—H3BA	120.0
C2A—C3A—H3AA	119.8	C4B—C3B—H3BA	120.0
C5A—C4A—C3A	119.95 (14)	C5B—C4B—C3B	120.13 (14)
C5A—C4A—H4AA	120.0	C5B—C4B—H4BA	119.9
C3A—C4A—H4AA	120.0	C3B—C4B—H4BA	119.9
C4A—C5A—C6A	120.02 (15)	C4B—C5B—C6B	120.12 (14)
C4A—C5A—H5AA	120.0	C4B—C5B—H5BA	119.9
C6A—C5A—H5AA	120.0	C6B—C5B—H5BA	119.9
C1A—C6A—C5A	120.39 (14)	C1B—C6B—C5B	119.93 (13)
C1A—C6A—H6AA	119.8	C1B—C6B—H6BA	120.0
C5A—C6A—H6AA	119.8	C5B—C6B—H6BA	120.0
C8A—C7A—C12A	119.20 (13)	C8B—C7B—C12B	119.57 (13)
C8A—C7A—As1A	122.40 (11)	C8B—C7B—As1B	120.75 (11)
C12A—C7A—As1A	118.18 (10)	C12B—C7B—As1B	119.22 (10)
C9A—C8A—C7A	120.24 (14)	C7B—C8B—C9B	119.95 (15)
C9A—C8A—H8AA	119.9	C7B—C8B—H8BA	120.0
C7A—C8A—H8AA	119.9	C9B—C8B—H8BA	120.0
C10A—C9A—C8A	120.54 (14)	C10B—C9B—C8B	120.52 (15)
C10A—C9A—H9AA	119.7	C10B—C9B—H9BA	119.7
C8A—C9A—H9AA	119.7	C8B—C9B—H9BA	119.7
C9A—C10A—C11A	119.50 (14)	C9B—C10B—C11B	119.56 (15)
C9A—C10A—H10A	120.3	C9B—C10B—H10B	120.2
C11A—C10A—H10A	120.3	C11B—C10B—H10B	120.2
C12A—C11A—C10A	120.29 (14)	C12B—C11B—C10B	120.33 (15)
C12A—C11A—H11A	119.9	C12B—C11B—H11B	119.8
C10A—C11A—H11A	119.9	C10B—C11B—H11B	119.8
C11A—C12A—C7A	120.23 (13)	C11B—C12B—C7B	120.06 (13)
C11A—C12A—H12A	119.9	C11B—C12B—H12B	120.0
C7A—C12A—H12A	119.9	C7B—C12B—H12B	120.0
As2A—C13A—As1A	110.18 (6)	As2B—C13B—As1B	111.93 (7)
As2A—C13A—H13A	109.6	As2B—C13B—H13C	109.2
As1A—C13A—H13A	109.6	As1B—C13B—H13C	109.2
As2A—C13A—H13B	109.6	As2B—C13B—H13D	109.2
As1A—C13A—H13B	109.6	As1B—C13B—H13D	109.2
H13A—C13A—H13B	108.1	H13C—C13B—H13D	107.9
C19A—C14A—C15A	119.37 (13)	C19B—C14B—C15B	119.88 (14)
C19A—C14A—As2A	123.08 (10)	C19B—C14B—As2B	123.26 (12)
C15A—C14A—As2A	117.45 (11)	C15B—C14B—As2B	116.72 (12)
C16A—C15A—C14A	120.40 (14)	C16B—C15B—C14B	120.07 (17)
C16A—C15A—H15A	119.8	C16B—C15B—H15B	120.0
C14A—C15A—H15A	119.8	C14B—C15B—H15B	120.0
C17A—C16A—C15A	119.87 (14)	C17B—C16B—C15B	119.63 (19)
C17A—C16A—H16A	120.1	C17B—C16B—H16B	120.2
C15A—C16A—H16A	120.1	C15B—C16B—H16B	120.2
C18A—C17A—C16A	120.07 (14)	C18B—C17B—C16B	120.38 (16)
C18A—C17A—H17A	120.0	C18B—C17B—H17B	119.8
C16A—C17A—H17A	120.0	C16B—C17B—H17B	119.8
C17A—C18A—C19A	120.28 (14)	C17B—C18B—C19B	120.30 (19)
C17A—C18A—H18A	119.9	C17B—C18B—H18B	119.8
C19A—C18A—H18A	119.9	C19B—C18B—H18B	119.8
C14A—C19A—C18A	120.00 (13)	C14B—C19B—C18B	119.70 (18)
C14A—C19A—H19A	120.0	C14B—C19B—H19B	120.1
C18A—C19A—H19A	120.0	C18B—C19B—H19B	120.1
C21A—C20A—C25A	119.66 (14)	C21B—C20B—C25B	119.71 (13)
C21A—C20A—As2A	118.43 (11)	C21B—C20B—As2B	120.78 (11)
C25A—C20A—As2A	121.91 (11)	C25B—C20B—As2B	119.48 (11)
C22A—C21A—C20A	120.19 (15)	C22B—C21B—C20B	120.10 (15)
C22A—C21A—H21A	119.9	C22B—C21B—H21B	119.9
C20A—C21A—H21A	119.9	C20B—C21B—H21B	119.9
C23A—C22A—C21A	120.07 (16)	C23B—C22B—C21B	120.21 (16)
C23A—C22A—H22A	120.0	C23B—C22B—H22B	119.9
C21A—C22A—H22A	120.0	C21B—C22B—H22B	119.9
C24A—C23A—C22A	120.01 (16)	C22B—C23B—C24B	119.85 (15)
C24A—C23A—H23A	120.0	C22B—C23B—H23B	120.1
C22A—C23A—H23A	120.0	C24B—C23B—H23B	120.1
C23A—C24A—C25A	120.20 (15)	C23B—C24B—C25B	120.28 (15)
C23A—C24A—H24A	119.9	C23B—C24B—H24B	119.9
C25A—C24A—H24A	119.9	C25B—C24B—H24B	119.9
C20A—C25A—C24A	119.85 (15)	C24B—C25B—C20B	119.84 (14)
C20A—C25A—H25A	120.1	C24B—C25B—H25B	120.1
C24A—C25A—H25A	120.1	C20B—C25B—H25B	120.1
O10A—C26A—C28A	106.24 (15)	O10B—C26B—C27B	108.21 (17)
O10A—C26A—C27A	110.15 (14)	O10B—C26B—C28B	107.62 (17)
C28A—C26A—C27A	113.22 (17)	C27B—C26B—C28B	112.35 (16)
O10A—C26A—H26A	109.0	O10B—C26B—H26B	109.5
C28A—C26A—H26A	109.0	C27B—C26B—H26B	109.5
C27A—C26A—H26A	109.0	C28B—C26B—H26B	109.5
C26A—C27A—H27A	109.5	C26B—C27B—H27D	109.5
C26A—C27A—H27B	109.5	C26B—C27B—H27E	109.5
H27A—C27A—H27B	109.5	H27D—C27B—H27E	109.5
C26A—C27A—H27C	109.5	C26B—C27B—H27F	109.5
H27A—C27A—H27C	109.5	H27D—C27B—H27F	109.5
H27B—C27A—H27C	109.5	H27E—C27B—H27F	109.5
C26A—C28A—H28A	109.5	C26B—C28B—H28D	109.5
C26A—C28A—H28B	109.5	C26B—C28B—H28E	109.5
H28A—C28A—H28B	109.5	H28D—C28B—H28E	109.5
C26A—C28A—H28C	109.5	C26B—C28B—H28F	109.5
H28A—C28A—H28C	109.5	H28D—C28B—H28F	109.5
H28B—C28A—H28C	109.5	H28E—C28B—H28F	109.5
O11A—C29A—C31A	106.39 (13)	O11B—C29B—C31B	108.60 (14)
O11A—C29A—C30A	108.61 (14)	O11B—C29B—C30B	106.35 (17)
C31A—C29A—C30A	112.14 (17)	C31B—C29B—C30B	112.28 (17)
O11A—C29A—H29A	109.9	O11B—C29B—H29B	109.8
C31A—C29A—H29A	109.9	C31B—C29B—H29B	109.8
C30A—C29A—H29A	109.9	C30B—C29B—H29B	109.8
C29A—C30A—H30A	109.5	C29B—C30B—H30D	109.5
C29A—C30A—H30B	109.5	C29B—C30B—H30E	109.5
H30A—C30A—H30B	109.5	H30D—C30B—H30E	109.5
C29A—C30A—H30C	109.5	C29B—C30B—H30F	109.5
H30A—C30A—H30C	109.5	H30D—C30B—H30F	109.5
H30B—C30A—H30C	109.5	H30E—C30B—H30F	109.5
C29A—C31A—H31A	109.5	C29B—C31B—H31D	109.5
C29A—C31A—H31B	109.5	C29B—C31B—H31E	109.5
H31A—C31A—H31B	109.5	H31D—C31B—H31E	109.5
C29A—C31A—H31C	109.5	C29B—C31B—H31F	109.5
H31A—C31A—H31C	109.5	H31D—C31B—H31F	109.5
H31B—C31A—H31C	109.5	H31E—C31B—H31F	109.5
O12A—C32A—C33A	108.69 (13)	O12B—C32B—C33B	109.34 (14)
O12A—C32A—C34A	106.76 (13)	O12B—C32B—C34B	106.40 (12)
C33A—C32A—C34A	113.43 (15)	C33B—C32B—C34B	112.50 (15)
O12A—C32A—H32A	109.3	O12B—C32B—H32B	109.5
C33A—C32A—H32A	109.3	C33B—C32B—H32B	109.5
C34A—C32A—H32A	109.3	C34B—C32B—H32B	109.5
C32A—C33A—H33A	109.5	C32B—C33B—H33D	109.5
C32A—C33A—H33B	109.5	C32B—C33B—H33E	109.5
H33A—C33A—H33B	109.5	H33D—C33B—H33E	109.5
C32A—C33A—H33C	109.5	C32B—C33B—H33F	109.5
H33A—C33A—H33C	109.5	H33D—C33B—H33F	109.5
H33B—C33A—H33C	109.5	H33E—C33B—H33F	109.5
C32A—C34A—H34A	109.5	C32B—C34B—H34D	109.5
C32A—C34A—H34B	109.5	C32B—C34B—H34E	109.5
H34A—C34A—H34B	109.5	H34D—C34B—H34E	109.5
C32A—C34A—H34C	109.5	C32B—C34B—H34F	109.5
H34A—C34A—H34C	109.5	H34D—C34B—H34F	109.5
H34B—C34A—H34C	109.5	H34E—C34B—H34F	109.5
O1A—C35A—Ru1A	171.58 (13)	O1B—C35B—Ru1B	174.05 (13)
O2A—C36A—Ru1A	177.84 (13)	O2B—C36B—Ru1B	179.52 (15)
O3A—C37A—Ru1A	172.82 (13)	O3B—C37B—Ru1B	173.51 (13)
O4A—C38A—Ru2A	175.30 (13)	O4B—C38B—Ru2B	173.16 (13)
O5A—C39A—Ru2A	176.23 (15)	O5B—C39B—Ru2B	176.98 (13)
O6A—C40A—Ru2A	172.77 (13)	O6B—C40B—Ru2B	173.57 (13)
O7A—C41A—Ru3A	173.30 (13)	O7B—C41B—Ru3B	174.79 (14)
O8A—C42A—Ru3A	178.35 (14)	O8B—C42B—Ru3B	175.89 (14)
O9A—C43A—Ru3A	172.86 (13)	O9B—C43B—Ru3B	174.57 (13)
			
C36A—Ru1A—Ru2A—C39A	67.2 (3)	C36B—Ru1B—Ru2B—C39B	−69.33 (19)
C37A—Ru1A—Ru2A—C39A	124.74 (14)	C35B—Ru1B—Ru2B—C39B	−138.77 (13)
C35A—Ru1A—Ru2A—C39A	−42.87 (14)	C37B—Ru1B—Ru2B—C39B	39.26 (13)
As1A—Ru1A—Ru2A—C39A	−141.84 (14)	As1B—Ru1B—Ru2B—C39B	130.64 (12)
Ru3A—Ru1A—Ru2A—C39A	31.40 (14)	Ru3B—Ru1B—Ru2B—C39B	−41.20 (12)
C36A—Ru1A—Ru2A—C40A	134.0 (2)	C36B—Ru1B—Ru2B—C38B	−132.67 (15)
C37A—Ru1A—Ru2A—C40A	−168.41 (6)	C35B—Ru1B—Ru2B—C38B	157.89 (6)
C35A—Ru1A—Ru2A—C40A	23.98 (6)	C37B—Ru1B—Ru2B—C38B	−24.08 (6)
As1A—Ru1A—Ru2A—C40A	−74.99 (4)	As1B—Ru1B—Ru2B—C38B	67.31 (5)
Ru3A—Ru1A—Ru2A—C40A	98.25 (4)	Ru3B—Ru1B—Ru2B—C38B	−104.54 (5)
C36A—Ru1A—Ru2A—C38A	−45.2 (2)	C36B—Ru1B—Ru2B—C40B	44.09 (15)
C37A—Ru1A—Ru2A—C38A	12.38 (6)	C35B—Ru1B—Ru2B—C40B	−25.35 (6)
C35A—Ru1A—Ru2A—C38A	−155.23 (6)	C37B—Ru1B—Ru2B—C40B	152.67 (6)
As1A—Ru1A—Ru2A—C38A	105.80 (4)	As1B—Ru1B—Ru2B—C40B	−115.94 (4)
Ru3A—Ru1A—Ru2A—C38A	−80.96 (4)	Ru3B—Ru1B—Ru2B—C40B	72.22 (4)
C36A—Ru1A—Ru2A—As2A	−142.0 (2)	C36B—Ru1B—Ru2B—As2B	138.51 (15)
C37A—Ru1A—Ru2A—As2A	−84.37 (4)	C35B—Ru1B—Ru2B—As2B	69.07 (4)
C35A—Ru1A—Ru2A—As2A	108.01 (4)	C37B—Ru1B—Ru2B—As2B	−112.91 (4)
As1A—Ru1A—Ru2A—As2A	9.043 (7)	As1B—Ru1B—Ru2B—As2B	−21.517 (6)
Ru3A—Ru1A—Ru2A—As2A	−177.713 (6)	Ru3B—Ru1B—Ru2B—As2B	166.638 (6)
C36A—Ru1A—Ru2A—Ru3A	35.8 (2)	C36B—Ru1B—Ru2B—Ru3B	−28.13 (15)
C37A—Ru1A—Ru2A—Ru3A	93.34 (4)	C35B—Ru1B—Ru2B—Ru3B	−97.57 (4)
C35A—Ru1A—Ru2A—Ru3A	−74.27 (4)	C37B—Ru1B—Ru2B—Ru3B	80.46 (4)
As1A—Ru1A—Ru2A—Ru3A	−173.243 (6)	As1B—Ru1B—Ru2B—Ru3B	171.845 (6)
C39A—Ru2A—Ru3A—C42A	17.12 (7)	C39B—Ru2B—Ru3B—C42B	−23.60 (7)
C40A—Ru2A—Ru3A—C42A	109.72 (7)	C38B—Ru2B—Ru3B—C42B	−116.45 (6)
C38A—Ru2A—Ru3A—C42A	−75.28 (7)	C40B—Ru2B—Ru3B—C42B	64.79 (6)
As2A—Ru2A—Ru3A—C42A	−167.34 (5)	As2B—Ru2B—Ru3B—C42B	143.93 (5)
Ru1A—Ru2A—Ru3A—C42A	−173.44 (5)	Ru1B—Ru2B—Ru3B—C42B	170.65 (5)
C39A—Ru2A—Ru3A—C43A	109.02 (7)	C39B—Ru2B—Ru3B—C41B	−116.55 (7)
C40A—Ru2A—Ru3A—C43A	−158.38 (6)	C38B—Ru2B—Ru3B—C41B	150.60 (7)
C38A—Ru2A—Ru3A—C43A	16.62 (6)	C40B—Ru2B—Ru3B—C41B	−28.17 (7)
As2A—Ru2A—Ru3A—C43A	−75.44 (5)	As2B—Ru2B—Ru3B—C41B	50.97 (5)
Ru1A—Ru2A—Ru3A—C43A	−81.54 (5)	Ru1B—Ru2B—Ru3B—C41B	77.69 (5)
C39A—Ru2A—Ru3A—C41A	−72.97 (7)	C39B—Ru2B—Ru3B—C43B	70.57 (6)
C40A—Ru2A—Ru3A—C41A	19.63 (6)	C38B—Ru2B—Ru3B—C43B	−22.28 (6)
C38A—Ru2A—Ru3A—C41A	−165.37 (6)	C40B—Ru2B—Ru3B—C43B	158.96 (6)
As2A—Ru2A—Ru3A—C41A	102.57 (5)	As2B—Ru2B—Ru3B—C43B	−121.90 (5)
Ru1A—Ru2A—Ru3A—C41A	96.47 (4)	Ru1B—Ru2B—Ru3B—C43B	−95.18 (4)
C39A—Ru2A—Ru3A—P1A	−122.82 (7)	C39B—Ru2B—Ru3B—P1B	127.65 (5)
C40A—Ru2A—Ru3A—P1A	−30.21 (7)	C38B—Ru2B—Ru3B—P1B	34.80 (5)
C38A—Ru2A—Ru3A—P1A	144.78 (6)	C40B—Ru2B—Ru3B—P1B	−143.96 (5)
As2A—Ru2A—Ru3A—P1A	52.72 (6)	As2B—Ru2B—Ru3B—P1B	−64.83 (3)
Ru1A—Ru2A—Ru3A—P1A	46.62 (5)	Ru1B—Ru2B—Ru3B—P1B	−38.11 (3)
C39A—Ru2A—Ru3A—Ru1A	−169.44 (5)	C39B—Ru2B—Ru3B—Ru1B	165.76 (5)
C40A—Ru2A—Ru3A—Ru1A	−76.83 (4)	C38B—Ru2B—Ru3B—Ru1B	72.91 (4)
C38A—Ru2A—Ru3A—Ru1A	98.16 (4)	C40B—Ru2B—Ru3B—Ru1B	−105.85 (4)
As2A—Ru2A—Ru3A—Ru1A	6.104 (16)	As2B—Ru2B—Ru3B—Ru1B	−26.720 (11)
C36A—Ru1A—Ru3A—C42A	−160.05 (11)	C36B—Ru1B—Ru3B—C42B	149.67 (12)
C37A—Ru1A—Ru3A—C42A	−69.60 (11)	C35B—Ru1B—Ru3B—C42B	54.35 (12)
C35A—Ru1A—Ru3A—C42A	116.76 (11)	C37B—Ru1B—Ru3B—C42B	−123.23 (12)
As1A—Ru1A—Ru3A—C42A	25.88 (10)	As1B—Ru1B—Ru3B—C42B	−39.62 (11)
Ru2A—Ru1A—Ru3A—C42A	12.94 (10)	Ru2B—Ru1B—Ru3B—C42B	−21.88 (11)
C36A—Ru1A—Ru3A—C43A	−72.77 (7)	C36B—Ru1B—Ru3B—C41B	69.08 (6)
C37A—Ru1A—Ru3A—C43A	17.68 (6)	C35B—Ru1B—Ru3B—C41B	−26.24 (6)
C35A—Ru1A—Ru3A—C43A	−155.96 (6)	C37B—Ru1B—Ru3B—C41B	156.18 (6)
As1A—Ru1A—Ru3A—C43A	113.15 (5)	As1B—Ru1B—Ru3B—C41B	−120.21 (5)
Ru2A—Ru1A—Ru3A—C43A	100.22 (4)	Ru2B—Ru1B—Ru3B—C41B	−102.47 (4)
C36A—Ru1A—Ru3A—C41A	104.41 (7)	C36B—Ru1B—Ru3B—C43B	−110.00 (6)
C37A—Ru1A—Ru3A—C41A	−165.14 (6)	C35B—Ru1B—Ru3B—C43B	154.68 (6)
C35A—Ru1A—Ru3A—C41A	21.22 (6)	C37B—Ru1B—Ru3B—C43B	−22.91 (6)
As1A—Ru1A—Ru3A—C41A	−69.67 (5)	As1B—Ru1B—Ru3B—C43B	60.71 (5)
Ru2A—Ru1A—Ru3A—C41A	−82.60 (5)	Ru2B—Ru1B—Ru3B—C43B	78.45 (4)
C36A—Ru1A—Ru3A—P1A	16.43 (5)	C36B—Ru1B—Ru3B—P1B	−22.22 (5)
C37A—Ru1A—Ru3A—P1A	106.88 (4)	C35B—Ru1B—Ru3B—P1B	−117.54 (4)
C35A—Ru1A—Ru3A—P1A	−66.76 (4)	C37B—Ru1B—Ru3B—P1B	64.88 (4)
As1A—Ru1A—Ru3A—P1A	−157.642 (14)	As1B—Ru1B—Ru3B—P1B	148.491 (15)
Ru2A—Ru1A—Ru3A—P1A	−170.574 (12)	Ru2B—Ru1B—Ru3B—P1B	166.232 (10)
C36A—Ru1A—Ru3A—Ru2A	−172.99 (5)	C36B—Ru1B—Ru3B—Ru2B	171.55 (4)
C37A—Ru1A—Ru3A—Ru2A	−82.55 (4)	C35B—Ru1B—Ru3B—Ru2B	76.23 (4)
C35A—Ru1A—Ru3A—Ru2A	103.81 (4)	C37B—Ru1B—Ru3B—Ru2B	−101.35 (4)
As1A—Ru1A—Ru3A—Ru2A	12.933 (11)	As1B—Ru1B—Ru3B—Ru2B	−17.741 (12)
C36A—Ru1A—As1A—C7A	64.92 (7)	C36B—Ru1B—As1B—C7B	72.03 (6)
C37A—Ru1A—As1A—C7A	−26.33 (6)	C35B—Ru1B—As1B—C7B	167.47 (6)
C35A—Ru1A—As1A—C7A	153.85 (6)	C37B—Ru1B—As1B—C7B	−16.41 (6)
Ru2A—Ru1A—As1A—C7A	−109.66 (5)	Ru3B—Ru1B—As1B—C7B	−98.78 (5)
Ru3A—Ru1A—As1A—C7A	−120.61 (5)	Ru2B—Ru1B—As1B—C7B	−114.00 (5)
C36A—Ru1A—As1A—C1A	−50.90 (7)	C36B—Ru1B—As1B—C1B	−51.31 (7)
C37A—Ru1A—As1A—C1A	−142.14 (6)	C35B—Ru1B—As1B—C1B	44.12 (6)
C35A—Ru1A—As1A—C1A	38.04 (6)	C37B—Ru1B—As1B—C1B	−139.76 (6)
Ru2A—Ru1A—As1A—C1A	134.53 (5)	Ru3B—Ru1B—As1B—C1B	137.87 (5)
Ru3A—Ru1A—As1A—C1A	123.57 (5)	Ru2B—Ru1B—As1B—C1B	122.65 (5)
C36A—Ru1A—As1A—C13A	−172.07 (7)	C36B—Ru1B—As1B—C13B	−170.23 (7)
C37A—Ru1A—As1A—C13A	96.68 (6)	C35B—Ru1B—As1B—C13B	−74.79 (6)
C35A—Ru1A—As1A—C13A	−83.14 (6)	C37B—Ru1B—As1B—C13B	101.32 (6)
Ru2A—Ru1A—As1A—C13A	13.35 (5)	Ru3B—Ru1B—As1B—C13B	18.95 (5)
Ru3A—Ru1A—As1A—C13A	2.39 (5)	Ru2B—Ru1B—As1B—C13B	3.74 (5)
C39A—Ru2A—As2A—C14A	25.81 (7)	C39B—Ru2B—As2B—C14B	106.20 (7)
C40A—Ru2A—As2A—C14A	−65.50 (6)	C38B—Ru2B—As2B—C14B	−162.75 (7)
C38A—Ru2A—As2A—C14A	120.54 (6)	C40B—Ru2B—As2B—C14B	13.86 (7)
Ru3A—Ru2A—As2A—C14A	−149.73 (5)	Ru3B—Ru2B—As2B—C14B	−61.11 (6)
Ru1A—Ru2A—As2A—C14A	−144.33 (5)	Ru1B—Ru2B—As2B—C14B	−84.02 (5)
C39A—Ru2A—As2A—C20A	−99.44 (7)	C39B—Ru2B—As2B—C20B	−15.31 (7)
C40A—Ru2A—As2A—C20A	169.26 (6)	C38B—Ru2B—As2B—C20B	75.74 (6)
C38A—Ru2A—As2A—C20A	−4.70 (6)	C40B—Ru2B—As2B—C20B	−107.64 (6)
Ru3A—Ru2A—As2A—C20A	85.03 (5)	Ru3B—Ru2B—As2B—C20B	177.38 (5)
Ru1A—Ru2A—As2A—C20A	90.43 (5)	Ru1B—Ru2B—As2B—C20B	154.48 (5)
C39A—Ru2A—As2A—C13A	139.85 (7)	C39B—Ru2B—As2B—C13B	−131.51 (6)
C40A—Ru2A—As2A—C13A	48.54 (6)	C38B—Ru2B—As2B—C13B	−40.46 (6)
C38A—Ru2A—As2A—C13A	−125.42 (6)	C40B—Ru2B—As2B—C13B	136.16 (6)
Ru3A—Ru2A—As2A—C13A	−35.69 (5)	Ru3B—Ru2B—As2B—C13B	61.18 (5)
Ru1A—Ru2A—As2A—C13A	−30.29 (5)	Ru1B—Ru2B—As2B—C13B	38.28 (5)
C42A—Ru3A—P1A—O11A	−116.15 (7)	C42B—Ru3B—P1B—O12B	127.58 (7)
C43A—Ru3A—P1A—O11A	151.78 (6)	C41B—Ru3B—P1B—O12B	−139.36 (7)
C41A—Ru3A—P1A—O11A	−26.61 (6)	C43B—Ru3B—P1B—O12B	32.98 (7)
Ru2A—Ru3A—P1A—O11A	23.09 (8)	Ru2B—Ru3B—P1B—O12B	−22.73 (6)
Ru1A—Ru3A—P1A—O11A	65.67 (5)	Ru1B—Ru3B—P1B—O12B	−56.05 (5)
C42A—Ru3A—P1A—O10A	−2.23 (7)	C42B—Ru3B—P1B—O11B	−116.72 (7)
C43A—Ru3A—P1A—O10A	−94.30 (7)	C41B—Ru3B—P1B—O11B	−23.67 (7)
C41A—Ru3A—P1A—O10A	87.30 (7)	C43B—Ru3B—P1B—O11B	148.68 (7)
Ru2A—Ru3A—P1A—O10A	137.01 (6)	Ru2B—Ru3B—P1B—O11B	92.97 (6)
Ru1A—Ru3A—P1A—O10A	179.58 (5)	Ru1B—Ru3B—P1B—O11B	59.65 (5)
C42A—Ru3A—P1A—O12A	117.14 (7)	C42B—Ru3B—P1B—O10B	5.80 (8)
C43A—Ru3A—P1A—O12A	25.07 (7)	C41B—Ru3B—P1B—O10B	98.85 (7)
C41A—Ru3A—P1A—O12A	−153.32 (7)	C43B—Ru3B—P1B—O10B	−88.80 (7)
Ru2A—Ru3A—P1A—O12A	−103.62 (7)	Ru2B—Ru3B—P1B—O10B	−144.51 (6)
Ru1A—Ru3A—P1A—O12A	−61.04 (5)	Ru1B—Ru3B—P1B—O10B	−177.83 (6)
O11A—P1A—O10A—C26A	179.62 (11)	O12B—P1B—O10B—C26B	−89.98 (14)
O12A—P1A—O10A—C26A	−70.77 (12)	O11B—P1B—O10B—C26B	166.78 (13)
Ru3A—P1A—O10A—C26A	60.09 (12)	Ru3B—P1B—O10B—C26B	33.39 (15)
O10A—P1A—O11A—C29A	33.16 (13)	O12B—P1B—O11B—C29B	−166.45 (12)
O12A—P1A—O11A—C29A	−68.72 (12)	O10B—P1B—O11B—C29B	−58.39 (13)
Ru3A—P1A—O11A—C29A	157.56 (10)	Ru3B—P1B—O11B—C29B	73.39 (13)
O11A—P1A—O12A—C32A	−79.79 (12)	O11B—P1B—O12B—C32B	50.58 (13)
O10A—P1A—O12A—C32A	176.79 (12)	O10B—P1B—O12B—C32B	−51.39 (13)
Ru3A—P1A—O12A—C32A	48.22 (13)	Ru3B—P1B—O12B—C32B	179.28 (11)
C7A—As1A—C1A—C6A	83.82 (13)	C7B—As1B—C1B—C6B	−132.60 (12)
C13A—As1A—C1A—C6A	−20.86 (14)	C13B—As1B—C1B—C6B	122.97 (12)
Ru1A—As1A—C1A—C6A	−149.85 (11)	Ru1B—As1B—C1B—C6B	−3.14 (13)
C7A—As1A—C1A—C2A	−89.09 (12)	C7B—As1B—C1B—C2B	52.11 (13)
C13A—As1A—C1A—C2A	166.23 (12)	C13B—As1B—C1B—C2B	−52.32 (13)
Ru1A—As1A—C1A—C2A	37.25 (13)	Ru1B—As1B—C1B—C2B	−178.43 (10)
C6A—C1A—C2A—C3A	−0.7 (2)	C6B—C1B—C2B—C3B	−1.5 (2)
As1A—C1A—C2A—C3A	172.34 (13)	As1B—C1B—C2B—C3B	173.85 (12)
C1A—C2A—C3A—C4A	0.9 (3)	C1B—C2B—C3B—C4B	0.0 (2)
C2A—C3A—C4A—C5A	0.2 (3)	C2B—C3B—C4B—C5B	1.5 (2)
C3A—C4A—C5A—C6A	−1.4 (3)	C3B—C4B—C5B—C6B	−1.4 (2)
C2A—C1A—C6A—C5A	−0.5 (2)	C2B—C1B—C6B—C5B	1.6 (2)
As1A—C1A—C6A—C5A	−173.35 (13)	As1B—C1B—C6B—C5B	−173.71 (11)
C4A—C5A—C6A—C1A	1.6 (3)	C4B—C5B—C6B—C1B	−0.1 (2)
C1A—As1A—C7A—C8A	−75.93 (13)	C1B—As1B—C7B—C8B	−145.53 (14)
C13A—As1A—C7A—C8A	25.92 (14)	C13B—As1B—C7B—C8B	−42.27 (15)
Ru1A—As1A—C7A—C8A	155.49 (11)	Ru1B—As1B—C7B—C8B	82.55 (14)
C1A—As1A—C7A—C12A	98.57 (12)	C1B—As1B—C7B—C12B	42.33 (13)
C13A—As1A—C7A—C12A	−159.59 (11)	C13B—As1B—C7B—C12B	145.60 (12)
Ru1A—As1A—C7A—C12A	−30.01 (13)	Ru1B—As1B—C7B—C12B	−89.59 (12)
C12A—C7A—C8A—C9A	0.3 (2)	C12B—C7B—C8B—C9B	−1.0 (3)
As1A—C7A—C8A—C9A	174.73 (12)	As1B—C7B—C8B—C9B	−173.15 (15)
C7A—C8A—C9A—C10A	−0.1 (3)	C7B—C8B—C9B—C10B	0.3 (3)
C8A—C9A—C10A—C11A	−0.2 (3)	C8B—C9B—C10B—C11B	0.6 (3)
C9A—C10A—C11A—C12A	0.3 (2)	C9B—C10B—C11B—C12B	−0.9 (3)
C10A—C11A—C12A—C7A	−0.1 (2)	C10B—C11B—C12B—C7B	0.1 (2)
C8A—C7A—C12A—C11A	−0.2 (2)	C8B—C7B—C12B—C11B	0.8 (2)
As1A—C7A—C12A—C11A	−174.87 (12)	As1B—C7B—C12B—C11B	173.03 (12)
C14A—As2A—C13A—As1A	165.10 (7)	C14B—As2B—C13B—As1B	88.38 (8)
C20A—As2A—C13A—As1A	−88.44 (8)	C20B—As2B—C13B—As1B	−168.92 (7)
Ru2A—As2A—C13A—As1A	43.52 (7)	Ru2B—As2B—C13B—As1B	−42.52 (8)
C7A—As1A—C13A—As2A	93.09 (7)	C7B—As1B—C13B—As2B	147.51 (7)
C1A—As1A—C13A—As2A	−168.20 (7)	C1B—As1B—C13B—As2B	−107.27 (8)
Ru1A—As1A—C13A—As2A	−36.83 (8)	Ru1B—As1B—C13B—As2B	22.10 (9)
C20A—As2A—C14A—C19A	−108.23 (13)	C20B—As2B—C14B—C19B	−107.22 (13)
C13A—As2A—C14A—C19A	−2.02 (13)	C13B—As2B—C14B—C19B	−3.33 (14)
Ru2A—As2A—C14A—C19A	116.80 (11)	Ru2B—As2B—C14B—C19B	122.33 (12)
C20A—As2A—C14A—C15A	75.49 (12)	C20B—As2B—C14B—C15B	68.55 (12)
C13A—As2A—C14A—C15A	−178.31 (12)	C13B—As2B—C14B—C15B	172.44 (11)
Ru2A—As2A—C14A—C15A	−59.49 (12)	Ru2B—As2B—C14B—C15B	−61.90 (12)
C19A—C14A—C15A—C16A	1.1 (2)	C19B—C14B—C15B—C16B	1.5 (2)
As2A—C14A—C15A—C16A	177.56 (13)	As2B—C14B—C15B—C16B	−174.46 (13)
C14A—C15A—C16A—C17A	−0.7 (3)	C14B—C15B—C16B—C17B	−1.0 (3)
C15A—C16A—C17A—C18A	−0.3 (3)	C15B—C16B—C17B—C18B	−0.5 (3)
C16A—C17A—C18A—C19A	0.8 (2)	C16B—C17B—C18B—C19B	1.5 (3)
C15A—C14A—C19A—C18A	−0.6 (2)	C15B—C14B—C19B—C18B	−0.5 (2)
As2A—C14A—C19A—C18A	−176.79 (11)	As2B—C14B—C19B—C18B	175.18 (13)
C17A—C18A—C19A—C14A	−0.4 (2)	C17B—C18B—C19B—C14B	−1.0 (3)
C14A—As2A—C20A—C21A	−174.38 (11)	C14B—As2B—C20B—C21B	18.73 (14)
C13A—As2A—C20A—C21A	79.94 (12)	C13B—As2B—C20B—C21B	−88.01 (14)
Ru2A—As2A—C20A—C21A	−44.27 (13)	Ru2B—As2B—C20B—C21B	150.28 (12)
C14A—As2A—C20A—C25A	5.91 (13)	C14B—As2B—C20B—C25B	−159.07 (12)
C13A—As2A—C20A—C25A	−99.77 (12)	C13B—As2B—C20B—C25B	94.18 (13)
Ru2A—As2A—C20A—C25A	136.02 (10)	Ru2B—As2B—C20B—C25B	−27.52 (14)
C25A—C20A—C21A—C22A	1.2 (2)	C25B—C20B—C21B—C22B	0.5 (3)
As2A—C20A—C21A—C22A	−178.53 (12)	As2B—C20B—C21B—C22B	−177.27 (14)
C20A—C21A—C22A—C23A	−1.3 (2)	C20B—C21B—C22B—C23B	−0.3 (3)
C21A—C22A—C23A—C24A	0.3 (3)	C21B—C22B—C23B—C24B	−0.4 (3)
C22A—C23A—C24A—C25A	0.8 (3)	C22B—C23B—C24B—C25B	0.9 (3)
C21A—C20A—C25A—C24A	−0.1 (2)	C23B—C24B—C25B—C20B	−0.7 (3)
As2A—C20A—C25A—C24A	179.66 (12)	C21B—C20B—C25B—C24B	0.0 (2)
C23A—C24A—C25A—C20A	−1.0 (2)	As2B—C20B—C25B—C24B	177.84 (13)
P1A—O10A—C26A—C28A	−147.83 (13)	P1B—O10B—C26B—C27B	116.73 (17)
P1A—O10A—C26A—C27A	89.19 (18)	P1B—O10B—C26B—C28B	−121.64 (15)
P1A—O11A—C29A—C31A	143.59 (14)	P1B—O11B—C29B—C31B	101.12 (17)
P1A—O11A—C29A—C30A	−95.51 (17)	P1B—O11B—C29B—C30B	−137.85 (13)
P1A—O12A—C32A—C33A	90.92 (14)	P1B—O12B—C32B—C33B	−98.61 (15)
P1A—O12A—C32A—C34A	−146.38 (12)	P1B—O12B—C32B—C34B	139.65 (13)
C36A—Ru1A—C35A—O1A	7.7 (9)	C36B—Ru1B—C35B—O1B	48.2 (12)
C37A—Ru1A—C35A—O1A	89.9 (10)	C37B—Ru1B—C35B—O1B	−174.1 (10)
As1A—Ru1A—C35A—O1A	−90.9 (9)	As1B—Ru1B—C35B—O1B	−54.2 (12)
Ru2A—Ru1A—C35A—O1A	177 (100)	Ru3B—Ru1B—C35B—O1B	153.4 (12)
Ru3A—Ru1A—C35A—O1A	121.2 (9)	Ru2B—Ru1B—C35B—O1B	−148.2 (12)
C37A—Ru1A—C36A—O2A	70 (4)	C35B—Ru1B—C36B—O2B	−100 (15)
C35A—Ru1A—C36A—O2A	−122 (4)	C37B—Ru1B—C36B—O2B	83 (15)
As1A—Ru1A—C36A—O2A	−24 (4)	As1B—Ru1B—C36B—O2B	−8(15)
Ru2A—Ru1A—C36A—O2A	127 (4)	Ru3B—Ru1B—C36B—O2B	168 (100)
Ru3A—Ru1A—C36A—O2A	160 (4)	Ru2B—Ru1B—C36B—O2B	−167 (100)
C36A—Ru1A—C37A—O3A	−21.1 (11)	C36B—Ru1B—C37B—O3B	−42.7 (12)
C35A—Ru1A—C37A—O3A	−103.0 (11)	C35B—Ru1B—C37B—O3B	179 (100)
As1A—Ru1A—C37A—O3A	77.8 (11)	As1B—Ru1B—C37B—O3B	59.5 (12)
Ru2A—Ru1A—C37A—O3A	168.2 (11)	Ru3B—Ru1B—C37B—O3B	−148.0 (12)
Ru3A—Ru1A—C37A—O3A	−133.8 (11)	Ru2B—Ru1B—C37B—O3B	153.9 (12)
C39A—Ru2A—C38A—O4A	42.3 (16)	C39B—Ru2B—C38B—O4B	49.3 (12)
C40A—Ru2A—C38A—O4A	−163.5 (14)	C40B—Ru2B—C38B—O4B	167.8 (9)
As2A—Ru2A—C38A—O4A	−59.1 (16)	As2B—Ru2B—C38B—O4B	−57.5 (12)
Ru3A—Ru2A—C38A—O4A	142.9 (16)	Ru3B—Ru2B—C38B—O4B	152.9 (11)
Ru1A—Ru2A—C38A—O4A	−156.3 (16)	Ru1B—Ru2B—C38B—O4B	−149.6 (12)
C40A—Ru2A—C39A—O5A	−6(2)	C38B—Ru2B—C39B—O5B	34 (3)
C38A—Ru2A—C39A—O5A	172 (2)	C40B—Ru2B—C39B—O5B	−142 (3)
As2A—Ru2A—C39A—O5A	−91 (2)	As2B—Ru2B—C39B—O5B	124 (3)
Ru3A—Ru2A—C39A—O5A	87 (2)	Ru3B—Ru2B—C39B—O5B	−63 (3)
Ru1A—Ru2A—C39A—O5A	59 (2)	Ru1B—Ru2B—C39B—O5B	−27 (3)
C39A—Ru2A—C40A—O6A	−62.4 (11)	C39B—Ru2B—C40B—O6B	−35.6 (12)
C38A—Ru2A—C40A—O6A	143.4 (9)	C38B—Ru2B—C40B—O6B	−154.0 (10)
As2A—Ru2A—C40A—O6A	38.4 (11)	As2B—Ru2B—C40B—O6B	71.4 (12)
Ru3A—Ru2A—C40A—O6A	−163.4 (11)	Ru3B—Ru2B—C40B—O6B	−139.0 (12)
Ru1A—Ru2A—C40A—O6A	136.1 (11)	Ru1B—Ru2B—C40B—O6B	163.9 (12)
C42A—Ru3A—C41A—O7A	46.8 (12)	C42B—Ru3B—C41B—O7B	44.8 (16)
C43A—Ru3A—C41A—O7A	−86.7 (18)	C43B—Ru3B—C41B—O7B	−153.8 (14)
P1A—Ru3A—C41A—O7A	−52.9 (12)	P1B—Ru3B—C41B—O7B	−59.9 (16)
Ru2A—Ru3A—C41A—O7A	136.4 (12)	Ru2B—Ru3B—C41B—O7B	140.0 (16)
Ru1A—Ru3A—C41A—O7A	−163.7 (12)	Ru1B—Ru3B—C41B—O7B	−160.7 (16)
C43A—Ru3A—C42A—O8A	−106 (6)	C41B—Ru3B—C42B—O8B	59 (2)
C41A—Ru3A—C42A—O8A	76 (6)	C43B—Ru3B—C42B—O8B	−119 (2)
P1A—Ru3A—C42A—O8A	163 (6)	P1B—Ru3B—C42B—O8B	152 (2)
Ru2A—Ru3A—C42A—O8A	−9(6)	Ru2B—Ru3B—C42B—O8B	−39 (2)
Ru1A—Ru3A—C42A—O8A	−21 (6)	Ru1B—Ru3B—C42B—O8B	−20 (2)
C42A—Ru3A—C43A—O9A	−57.8 (12)	C42B—Ru3B—C43B—O9B	−66.2 (15)
C41A—Ru3A—C43A—O9A	75.7 (19)	C41B—Ru3B—C43B—O9B	132.3 (13)
P1A—Ru3A—C43A—O9A	42.0 (12)	P1B—Ru3B—C43B—O9B	38.2 (15)
Ru2A—Ru3A—C43A—O9A	−147.6 (12)	Ru2B—Ru3B—C43B—O9B	−160.3 (15)
Ru1A—Ru3A—C43A—O9A	152.9 (12)	Ru1B—Ru3B—C43B—O9B	139.2 (15)

### Hydrogen-bond geometry (Å, °)

**Table:**

*D*—H···*A*	*D*—H	H···*A*	*D*···*A*	*D*—H···*A*
C10B—H10B···O6A^i^	0.93	2.59	3.293 (2)	132
C17B—H17B···O2B^ii^	0.93	2.57	3.477 (2)	165
C33A—H33B···O2A^iii^	0.96	2.56	3.492 (2)	164

Symmetry codes: (i) −*x*, −*y*+1, −*z*; (ii) −*x*+1, −*y*+1, −*z*; (iii) −*x*, −*y*+1, −*z*+1.
